# Porphyrin-Based Compounds in Medicine: From Photodynamic Therapy to Theranostics

**DOI:** 10.3390/molecules31193488

**Published:** 2026-10-01

**Authors:** Urszula Rodzoń-Wańczura, David Aebisher, Tamil Selvan Ramesh, Klaudia Dynarowicz, Maciej Filip, Dorota Bartusik-Aebisher

**Affiliations:** 1 Department of Interventional Radiology, Electrophysiology and Electrotherapy, The Ministry of Internal Affairs and Administration Hospital, 35-111 Rzeszów, Poland; urodzonwanczura@gmail.com; 2 Department of Photomedicine and Physical Chemistry, Faculty of Medicine, University of Rzeszów, 35-310 Rzeszów, Poland; 3 Medical Science Club, Faculty of Medicine, University of Rzeszów, 35-310 Rzeszów, Poland; tr131820@stud.ur.edu.pl (T.S.R.); mf132718@stud.ur.edu.pl (M.F.); 4 Department of Biochemistry and General Chemistry, Faculty of Medicine, University of Rzeszów, 35-310 Rzeszów, Poland; kdynarowicz@ur.edu.pl (K.D.); dbartusikaebisher@ur.edu.pl (D.B.-A.)

**Keywords:** porphyrins, photosensitizers, photodynamic therapy, theranostics, nanomedicine

## Abstract

Porphyrins and their derivatives represent one of the most adaptable classes of compounds in contemporary biomedical research, bridging the crucial gap between diagnostic imaging and therapeutic intervention. This comprehensive review examines the evolution of porphyrin-based compounds and their therapeutic applications, from their fundamental role in photodynamic therapy to their status as sophisticated theranostic platforms. The unique photophysical properties of porphyrins, which are characterized by intense Soret band absorption, tunable fluorescence emission, and the generation of reactive oxygen species upon light activation, underpin their dual functionality as both imaging and therapeutic agents. The structural basis of porphyrin photochemistry, their progression through the first, second, and third generation of photosensitizers, and the transformative impact of nanotechnology, especially in overcoming intrinsic limitations such as poor aqueous solubility, aggregation-caused quenching, as well as limited tumor specificity, are discussed. Attention is devoted to emerging nanoplatforms such as porphyrin-based metal–organic frameworks (MOFs), covalent–organic frameworks (COFs), and electrospun nanofibers, which enable multimodal imaging, synergistic therapies, and controlled drug delivery. The challenges that impede clinical translation, including phototoxicity, tissue penetration limitations, and biosafety concerns, are critically examined, along with promising strategies for their resolution. By integrating porphyrin-based theranostic capabilities within single molecular platforms, these systems are ready to advance personalized cancer medicine while enabling real-time treatment monitoring and precision-guided interventions, which could substantially improve patient outcomes.

## 1. Introduction

Porphyrins are a group of macrocyclic organic compounds built from four modified pyrrole subunits connected by methine bridges [1,2,3]. They are a class of pigments often distinguished by the type of metal ion at their core [4]. They act as pigments of life, forming vital metal complexes such as heme (iron) and chlorophyll (magnesium) [5,6]. Due to their conjugated ring system, porphyrins absorb light intensely at around 400 nm and emit a strong red fluorescence [6,7]. In humans, abnormalities in heme biosynthesis can cause porphyrins to accumulate in the body [8]. Physicians evaluate heme production by measuring levels of specific porphyrins (uroporphyrins, coproporphyrin, and protoporphyrin) through blood and urine tests [9,10,11,12]. Because of their highly tunable redox and optical properties, synthetic and modified porphyrins are widely investigated outside of nature. In particular, they are used as photosensitizers in photodynamic therapy (PDT) for certain cancers, as well as in solar energy conversion, gas detection, and molecular electronics [13,14,15,16,17,18].

Cancer remains one of the most formidable challenges in medicine, with the International Agency for Research on Cancer reporting 20 million new cases in 2022, with projections indicating a rise to over 35 million new cases by 2050 [19,20]. Conventional therapies such as surgery, chemotherapy, and radiation therapy are frequently restricted by limited tumor specificity, therapeutic resistance, and systemic toxicity. Such limitations have catalyzed intensive research into alternative strategies [21,22,23].

Photodynamic therapy has emerged as a promising minimally invasive approach that selectively targets tumor cells while preserving normal tissues [24,25]. The therapeutic mechanism involves the administration of a photosensitizing agent that accumulates in tumor cells, followed by localized irradiation with light of an appropriate wavelength [26]. This triggers photochemical reactions that produce reactive oxygen species (ROS), resulting in targeted cellular destruction [27]. Photodynamic therapy is especially valuable for treating various malignancies, such as skin, esophageal, colorectal, lung, head and neck, and early-stage prostate cancers. Among the diverse classes of photosensitizing agents, porphyrins and their derivatives occupy a singular importance in PDT [28,29,30,31].

The porphyrin macrocycle has an aromatic 18-π-electron conjugated pathway within a 22-π-electron system, consistent with Hückel’s (4n + 2) rule [17,32]. This extended π-electron conjugation contributes to the characteristic spectral and photophysical properties of porphyrins, including strong absorption in the visible region, fluorescence, efficient intersystem crossing to the triplet state, and singlet oxygen generation upon light activation [33,34]. However, tumor selectivity and low dark toxicity are biological and pharmacological properties that depend on the molecular structure, cellular uptake, tissue distribution, and pharmacokinetic behavior of individual photosensitizers rather than directly resulting from the 18-π-electron aromatic system. Porphyrin-based photosensitizers have evolved over three generations, with each generation addressing the limitations of its predecessors. The first-generation photosensitizers, represented by hematoporphyrin derivatives (HpD) and Photofrin, established the clinical proof of concept for PDT [13,25]. However, they suffered from limitations such as suboptimal light absorption, prolonged skin photosensitivity, and heterogeneous composition [25]. Benzoporphyrin derivatives and protoporphyrin IX from 5-aminolevulinic acid (ALA) are second-generation photosensitizers that offer reduced photosensitivity and improved photophysical properties [13,25,35]. The third-generation photosensitizers—the current frontier—integrate porphyrins with nanotechnology, ligands (tumor targeting), and diagnostic imaging capabilities to create improved theranostic platforms. This integration simultaneously enables disease detection, treatment, and therapeutic monitoring [25,35].

The unification of nanotechnology and porphyrin chemistry has catalyzed a paradigm shift in cancer management. Either encapsulating porphyrins within nanoscale frameworks or incorporation into nanoscale frameworks resulted in substantial improvements in photostability, aqueous solubility, tumor targeting, and pharmacokinetic profiles. The nanoscale frameworks include metal–organic frameworks (MOFs), covalent–organic frameworks (COFs), liposomes, electrospun nanofibers, and polymeric nanoparticles. Such advances have enabled the realization of the concept of ‘theranostics’, which integrates diagnostic and therapeutic functionalities within a single nanoplatform. This allows real-time assessment of tumor location and response to treatment [36,37,38,39,40].

Although many current studies address porphyrin photophysics and chemistry or porphyrin-based photosensitizers or nanoplatforms such as porphyrin-MOFs, many combine the whole range of new nanoarchitectures, including MOFs, COFs, and electrospun nanofibers, with different therapeutic methods (photothermal, sonodynamic, chemodynamic, and immunotherapeutic) and multiple imaging tools in one clear translation-focused comparison. Electrospun nanofiber platforms for delivering porphyrins are still rarely reviewed in the literature, even though they are operable for local and long-term release. This review addresses that gap by following how porphyrin-based photosensitizers have changed from one generation to the next, looking at their status in clinical translation, critically weighing the strengths and limitations of MOF, COF, and nanofiber delivery systems, and combining therapy and imaging approaches inside the theranostic framework while identifying the barriers that continue to impede their clinical translation.

## 2. Materials and Methods

A thorough literature search was conducted from 15 April to 31 July 2026 using electronic medical databases and supplementary sources, such as PubMed, Scopus, Google Scholar, Research Gate, and other sources. Additional records were also retrieved from health websites, conference abstracts, and hand searching reference lists of included reviews and key original articles. The following search string was used in each database: (1) porphyrins, and/or (2) photodynamic therapy, and/or (3) theranostics, and/or (4) combination therapy/novel strategies, and/or (5) chemistry, and/or (6) photosensitizer and/or (7) nanomedicine. The database-specific syntax was adapted when necessary. Inclusion criteria were peer-reviewed original research, clinical trials, experimental studies, reviews, book chapters, dissertation, preprints, commercial/industrial product monographs, and governmental/online websites. Articles published in English within the last 35 years (published between 1991 and May 2026) with exception of two older references, studies focusing on porphyrin chemistry and fundamental properties, photodynamic therapy, photosensitizers, nanomedicine and drug delivery systems, theranostics and imaging, combination therapy strategies, along with clinical and medical applications, were given priority. Exclusion criteria included non-English publications, articles older than 35 years (with the exception of three references), non-medical applications, non-porphyrin photosensitizers, retracted articles, duplicate records, and studies with unavailable full text due to institutional access restrictions. A key limitation of this study was restricted institutional access to certain journals and databases, which may have limited retrieval of some full-text articles. The remaining 209 full-text records were retrieved, and all met eligibility criteria for inclusion. Schematic and mechanistic figures were hand-drawn using Microsoft Word version 2602 and GoodNotes version 5. Chemical structures were generated by the authors based on canonical structures retrieved from PubChem (National Center for Biotechnology Information) and rendered using MolView-assessed on 11 September 2026.

## 3. Structural and Photophysical Foundations of Porphyrins

### 3.1. Molecular Architecture

The porphyrin macrocycle consists of four pyrrole subunits, which are interconnected by four methine bridges (-CH=) at positions (5, 10, 15, 20), making the arrangement a highly conjugated, planar tetrapyrrolic ring system with a central cavity [32]. This cavity coordinates with a wide range of metal ions. The core structure exhibits remarkable aromaticity, with 22 π-electrons distributed throughout the macrocycle [32,41]. The structural versatility of porphyrins enables extensive chemical modification through substitution at the β-pyrrolic or meso positions [42]. These derivatizations allow for precise tuning of photophysical properties, solubility, and biological interactions [43]. The introduction of functional groups such as sulfonate, carboxylate, pyridyl, and glycosyl moieties can substantially enhance aqueous solubility and further facilitate conjugation with targeting ligands or imaging probes [44].

### 3.2. Photophysical Properties and ROS Generation

In porphyrins, the electronic absorption spectrum is characterized by an intense B-band (Soret band) centered between 400–450 nm, corresponding to π → π* transitions to the second excited singlet state (S_2_) [3,14]. It is accompanied by four less intense Q bands in the 500–700 nm range, which represent transitions to the first excited singlet state (S_1_) [14,34,45]. However, the relative intensities and positions of these absorption bands are influenced by factors such as molecular symmetry, metal coordination, the nature and position of peripheral substituents and protonation state [46].

**Figure 1:**
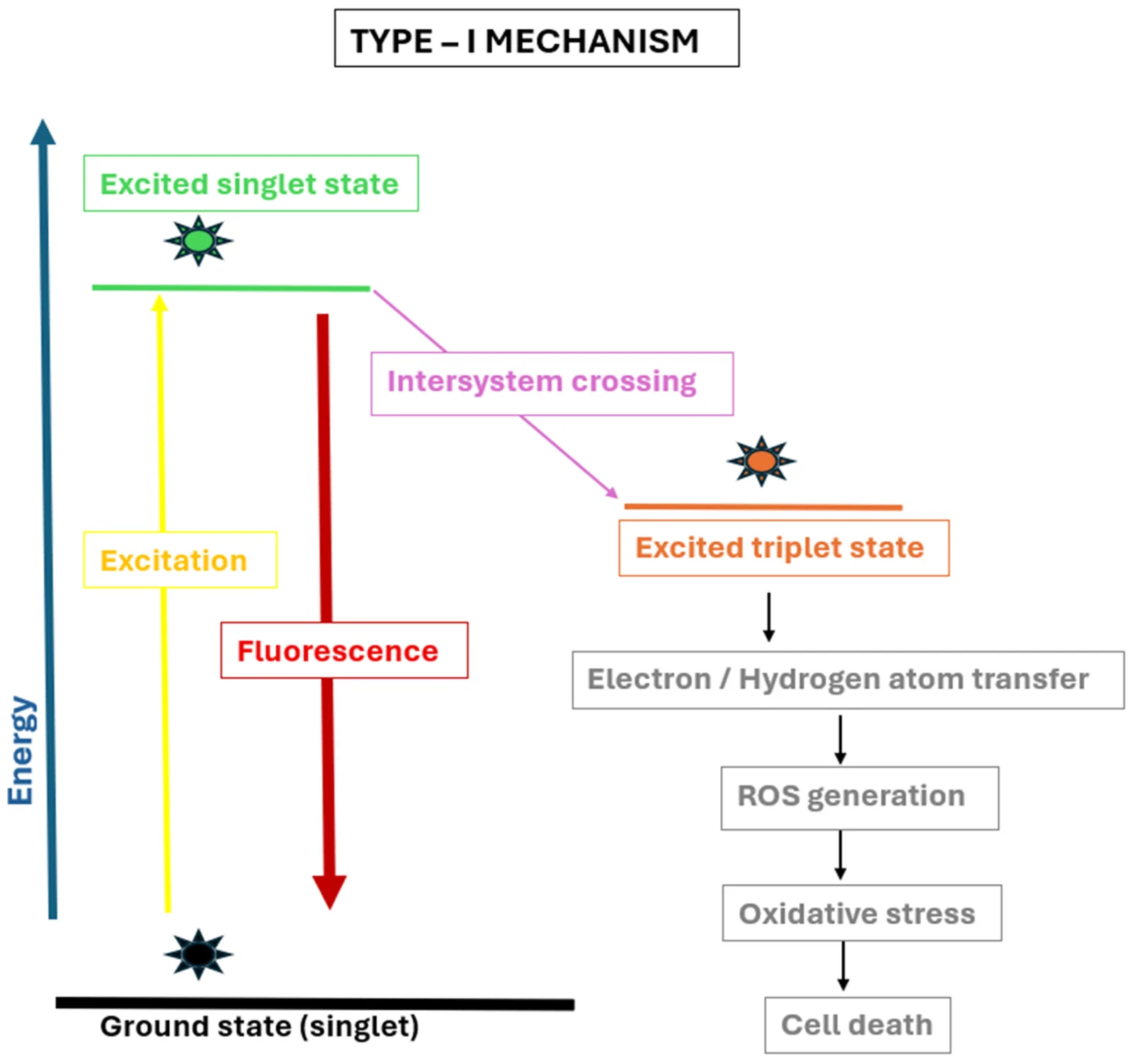
Type I photodynamic mechanism. The schematic figure illustrates the Type I photodynamic mechanism, beginning with a photosensitizer (star) in its ground singlet state (black). Upon excitation, it absorbs light energy (blue) to transition to an excited singlet state (green). From this short-lived state, the molecule may either decay back to the ground state via fluorescence emission (red) or undergo intersystem crossing (purple) a non-radiative process to reach the longer-lived excited triplet state (orange). In Type I pathway, this reactive triplet state drives electron or hydrogen atom transfer to surrounding biological substrates, subsequently generating reactive oxygen species (ROS) such as superoxide and hydroxyl radicals. The accumulation of these species induces oxidative stress within the cellular environment, ultimately leading to cell death as the final biological outcome. Thus, the cascade follows: excitation → excited singlet → (fluorescence) ↔ intersystem crossing→ excited triplet → electron/hydrogen atom transfer → ROS generation → oxidative stress → cell death.

**Figure 2:**
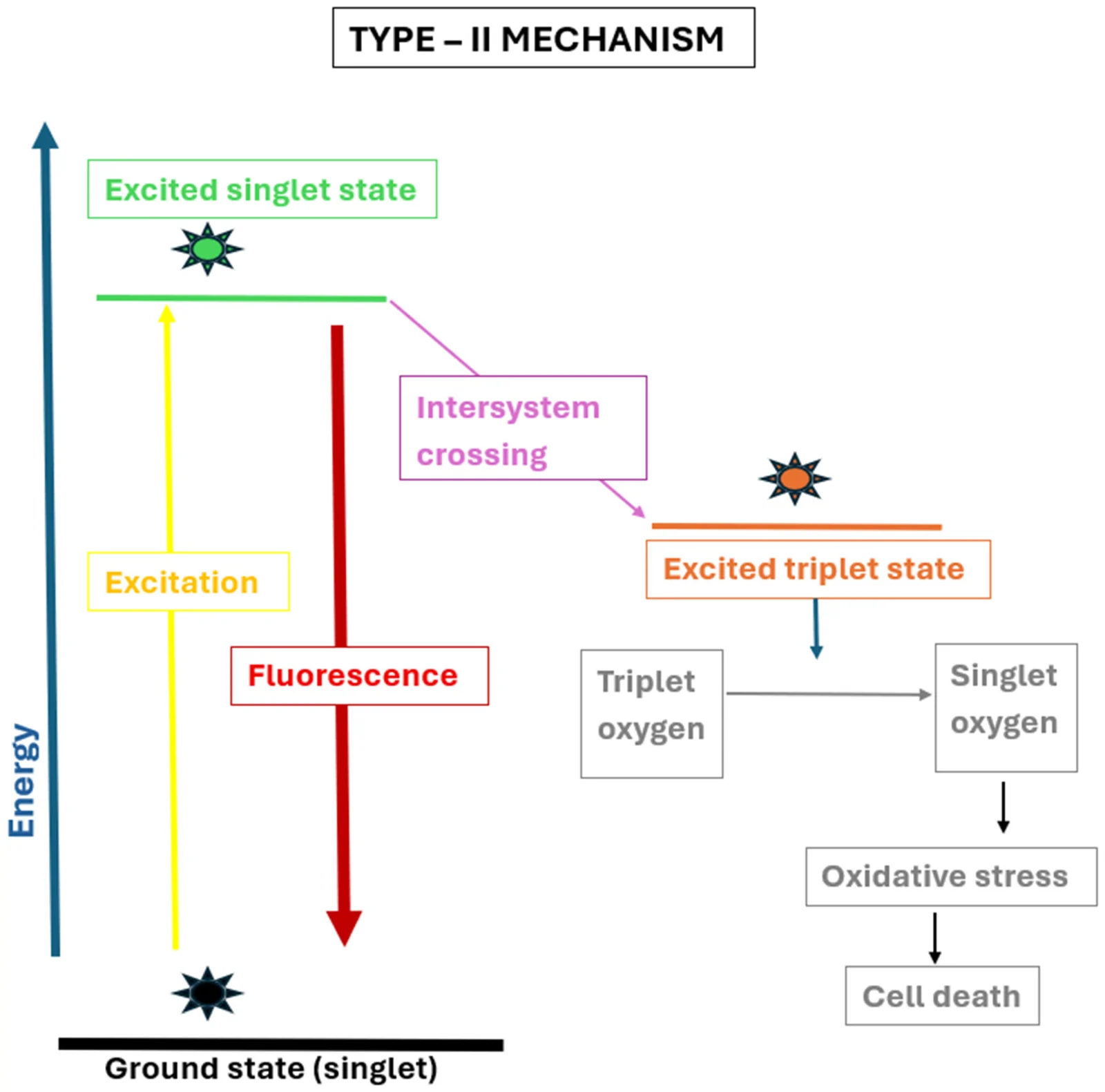
Type II photodynamic mechanism. The schematic figure illustrates the Type II photodynamic mechanism, beginning with a photosensitizer (star) in its ground singlet state (black). Upon excitation, it absorbs light energy (blue) to transition to an excited singlet state (green). From this short-lived state, the molecule may either decay back to the ground state via fluorescence emission (red) or undergo intersystem crossing (purple) a non-radiative process to reach the longer-lived excited triplet state (orange). In the Type II pathway, this reactive triplet state directly transfers its energy to molecular triplet oxygen (^3^O_2_), promoting it to highly reactive singlet oxygen (^1^O_2_). The generation of this cytotoxic singlet oxygen species subsequently induces oxidative stress within the cellular environment, which ultimately leads to cell death as the final biological outcome. Thus, the cascade follows: excitation → excited singlet → (fluorescence) ↔ intersystem crossing→ excited triplet → energy transfer to oxygen → singlet oxygen generation → oxidative stress → cell death.

In PDT, the photochemical cascade begins with photon absorption, promoting the porphyrin from its ground singlet state (S_0_) to its excited singlet state (S_1_ or S_2_). Following internal conversion and vibrational relaxation, the molecule (porphyrin) may return the ground state through fluorescence emission or by undergoing intersystem crossing to a longer-lived excited triplet state (T_1_). The lifetime of this triplet state can range from microseconds to milliseconds [47]. It can participate in two distinct photochemical pathways, namely Type I and Type II [2,25,47]. In the Type I mechanism, electron or hydrogen transfer from the triplet state photosensitizer to neighboring substrates such as water, oxygen, or biomolecules. This results in generation of hydrogen peroxide (H_2_O_2_) and radical species including superoxide anion (·O_2_^−^), and hydroxyl radical (·OH) [25,48] (Figure 1 and Figure 2).

The Type II pathway involves the direct transfer of energy from the photosensitizer in its excited triplet state to ground-state molecular oxygen (^3^O_2_), leading to the formation of singlet oxygen (^1^O_2_) and the return of the photosensitizer to its ground state [25,48,49,50,51,52]. Singlet oxygen is, thus, a characteristic reactive oxygen species (ROS) associated with Type II photochemistry. Due to its short lifetime and limited diffusion range, ^1^O_2_ typically causes oxidative damage in the immediate vicinity of the photosensitizer’s intracellular or subcellular location [47,53,54].

The distinction between Type I and Type II pathways can be experimentally investigated by combining the use of ROS-specific probes, chemical quenchers, and spectroscopic methods. Singlet oxygen production is commonly assessed using probes such as Singlet Oxygen Sensor Green (SOSG) [55], 1,3-diphenylisobenzofuran (DPBF), or through the direct detection of characteristic ^1^O_2_ luminescence at a wavelength of approximately 1270–1280 nm [56]. In contrast, ROS associated with the Type I mechanism can be studied using probes such as dihydroethidium (DHE) or related probes for the detection of superoxide anion [57], and Amplex Red or related assays for the determination of hydrogen peroxide [58]. Chemical quenchers can also be used to confirm the reaction mechanism; for example, sodium azide is commonly employed as a singlet oxygen quencher, while catalase and superoxide dismutase can serve to modulate pathways dependent on H_2_O_2_ and the superoxide radical anion, respectively [59].

Importantly, no single ROS probe should be considered sufficient to unequivocally determine the PDT mechanism, as many fluorescent probes lack complete specificity and may undergo secondary oxidation reactions. Therefore, employing a combination of complementary detection methods provides more reliable evidence for distinguishing between Type I and Type II photochemistry.

The generated ROS cause oxidative damage upon cellular components, including proteins, lipids, and nucleic acids, triggering diverse cell death pathways, such as apoptosis, necrosis, enzyme mediated apoptosis and autophagy [51,52]. The short lifetime of singlet oxygen (approximately 3.6 μs in water, note: inside a living cell, its lifetime drops drastically to about 0.04 to 0.2 μs due to immediate quenching by high densities of intracellular biomolecules like proteins and lipids) and limited diffusion radius (0.01–0.55 μm) confines oxidative damage to the immediate vicinity of the photosensitizer’s location. This underscores the importance of subcellular targeting [47,53,54].

### 3.3. Fluorescence Properties and Imaging Applications

In addition to ROS generation, porphyrins exhibit intrinsic fluorescence, rendering them a valuable imaging agent [60,61]. Usually, fluorescence emission occurs between 650 and 750 nm. However, the exact wavelength is dependent on substituent patterns, metal coordination, and environmental conditions [62,63]. This emission falls within the near-infrared optical window where tissue autofluorescence is minimal and photon penetration is optimal, making porphyrins suitable for fluorescence-guided surgery and tumor visualization [64,65,66].

The fluorescence properties of porphyrins are influenced by molecular aggregation, a phenomenon which has significant implications for both imaging and therapeutic applications [67]. In aqueous environments, porphyrins tend to aggregate via π-π stacking interactions, forming J-type or H-type aggregates, exhibiting characteristic bathochromic or hypsochromic spectral shifts, often displaying reduced fluorescence quantum yields and reduced ROS generation efficiency. This aggregation-induced quenching represents a major limitation and has driven extensive research to maintain porphyrin monomeric dispersion, including encapsulation within nanocarriers and covalent incorporation into framework materials [32,68,69,70].

## 4. Evolution of Porphyrin-Based Photosensitizers

### 4.1. First-Generation Photosensitizers

Porphyrins as photosensitizing agents were recognized in the early 20th century in 1942, with Auler and Banzer demonstrating that hematoporphyrin (Hp) accumulates in animal tumors and that such tumors are eliminated upon light exposure [71,72]. Following that, nearly two decades later, the 1960s marked the beginning of PDT’s evolution as a legitimate diagnostic and therapeutic modality, with hematoporphyrin derivative (HpD) serving as the prototypical photosensitizer [71,72,73]. Hematoporphyrin derivative was obtained by chemical modification of Hp and showed improvements in tumor selectivity for tumors and reduced photosensitizing potential on the skin. Low chemical purity, long half-life and its accumulation in the skin, and poor tissue penetration are some of its limitations in clinical applications [25].

### 4.2. Second-Generation Photosensitizers

The limitations of first-generation photosensitizing agents motivated the development of second-generation photosensitizers. They are characterized by improved photophysical properties, enhanced tumor selectivity, and reduced dark toxicity while maintaining effective phototoxic activity upon light irradiation. Limitations associated with the use of first-generation photosensitizers drove the development of second-generation agents. Within this classification, second-generation agents are distinguished primarily by superior photophysical and pharmacological properties compared to first-generation preparations; these include, among others, stronger absorption of radiation at longer [74] therapeutic wavelengths, higher photochemical efficiency, greater selectivity for tumor tissue, more favorable pharmacokinetics, and reduced toxicity in the absence of irradiation (so-called “dark toxicity”) [75]. These improvements were achieved mainly through chemical and structural modifications of porphyrin and porphyrinoid compounds. Key compounds include ALA (a porphyrin precursor) and a benzoporphyrin derivative, benzoporphyrin derivative monoacid ring A (BPD-MA, verteporfin). Other groups of compounds include texaphyrins, chlorins, bacteriochlorin analogs, and phthalocyanines [32].

ALA exploits the heme biosynthesis pathway to induce endogenous accumulation of protoporphyrin IX in malignant tissues, whereas BPD-MA exhibits strong absorption in the red region (690 nm) and favorable pharmacokinetics [76,77,78,79].

Second-generation porphyrinoids including chlorins, phthalocyanines, and bacteriochlorins offer extended absorption into the near-infrared region (650–800 nm). This allows enhanced tissue penetration due to reduced scattering and absorption by water and hemoglobins. As such, the improved photophysical properties of these compounds facilitate deeper tumor treatment and reduce cutaneous photosensitivity. However, despite advances, second-generation agents face challenges such as toxicity-induced tissue damage and low stabilities [80,81].

### 4.3. Third-Generation Photosensitizers and the Rise of Theranostics

Third-generation photosensitizers are distinguished from the second generation by the incorporation of active targeting, advanced delivery, or multifunctional components designed to further improve selective tumor localization, biodistribution, and therapeutic monitoring [82,83].

Third-generation photosensitizing agents are the current frontier of photosensitizer development, which seeks to address the limitations of previous-generation compounds through molecular design and nanotechnology integration. These systems typically incorporate multiple functional elements, including targeting ligands (antibodies, folate, peptides, or carbohydrates), nanocarriers (liposomes, nanoparticles, micelles, MOFs, and COFs), imaging contrast agents, and drug delivery capacity. Targeting ligands enhance tumor specificity and reduce off-target effects; nanocarriers improve solubility, stability, and pharmacokinetic profiles; imaging contrast agents enable real-time therapeutic monitoring and drug delivery capacity for combination chemotherapy. The integration of therapeutic and diagnostic capabilities within a single nanoplatform, the theranostic paradigm, represents the most significant advancement in third-generation photosensitizer design. By simultaneous monitoring imaging, therapy, and treatment, it facilitates personalized medicine approaches, in which therapeutic interventions can be performed based on real-time feedback on tumor burden, drug distribution, and treatment response [25,81,82,83,84,85,86,87,88,89,90,91,92,93,94,95,96].

Despite these advantages, the integration of nanocarriers and multifunctional components also presents a range of challenges that may limit the clinical implementation of third-generation photosensitizers. Potential problems include nanocarrier-related toxicity, long-term biological safety, accumulation and clearance from the body, and possible interactions with biological systems, which can vary depending on the composition, size, surface properties, and degradation profile of the nanoparticles [97]. Furthermore, ensuring batch-to-batch reproducibility and controlling physicochemical properties can pose challenges for large-scale production, potentially affecting photosensitizer loading, stability, biodistribution, and therapeutic efficacy [98]. Therefore, while nanotechnology-based platforms offer enhanced targeting and multifunctionality, their clinical development requires a thorough evaluation of long-term safety, pharmacokinetics, manufacturing reproducibility, and regulatory requirements [99].

## 5. Nanotechnological Strategies for Porphyrin Delivery and Performance Enhancement

Even with their remarkable photophysical properties, the clinical application is constrained by several interrelated challenges. These challenges include poor aqueous solubility (the hydrophobic character of porphyrin macrocycles leads to aggregation in biological environments), quenching due to aggregation (π-π stacking between porphyrin molecules, which dramatically reduces fluorescence emission and ROS generation efficiency), limited tissue penetration (absorption in the visible light spectrum restricts therapeutic depth to less than 2 cm), insufficient tumor specificity (passive accumulation through the effect of enhanced permeability and retention (EPR) provides limited selectivity), rapid clearance and metabolism (short circulation half-life reducing tumor accumulation), and potential phototoxicity (nonspecific activation can damage healthy tissues) [100].

Nanotechnology-based approaches have emerged as the most promising strategy for addressing these challenges. Diverse platforms have been developed to enhance porphyrin solubility, stability, and targeting while enabling multimodal functionality.

### 5.1. Porphyrin-Based MOFs

MOFs represent one of the most flexible strategies for porphyrin delivery and therapeutic applications. They are crystalline porous materials that are constructed from metal ions or clusters connected by organic linkers. They offer extraordinary structural tunability, ultra-high porosity, and vast surface areas [101]. Porphyrin-based MOFs can be categorized into two principal classes, namely porphyrin@MOFs and porphyrinic MOFs [102,103].

This was achieved while maintaining molecular dispersion [104]. Spatial confinement within the ZIF-8 matrix was shown to keep Ce6 molecules isolated and prevent self-aggregation [105]. However, it is important to note that for porphyrin@MOF systems, premature leakage and self-quenching of porphyrins remain recognized challenges that require formulation-specific monitoring during synthesis.

In porphyrinic MOFs, the porphyrins are directly integrated into the MOF framework as organic ligands, coordinating with metal ions or clusters. Such coordination incorporation ensures uniform distribution of porphyrin [102]. The porphyrin coordination network (PCN-224) is an example widely studied, with zirconium clusters connected by tetrakis (4-carboxyphenyl) porphyrin ligands [106]. Formulation-specific evidence demonstrates that framework incorporation can prevent aggregation. For example, in Fe(TCPP)-MOF, the porphyrin molecules were precisely oriented and firmly confined within the scaffold, with a distance of 6.8 Å between two porphyrin molecules, confirming the absence of π–π stacking interactions and thereby avoiding ACQ [107].

Porphyrin-based MOFs offer numerous distinctive advantages for theranostic applications, such as enhanced photodynamic activity (framework incorporation prevents ACQ, while maintaining or enhancing singlet oxygen generation efficiency), multimodal imaging (integration of various metal ions enables MRI, PTI, PAI, or CT contrast), high drug loading capacity (porous structure facilitates co-loading of chemotherapeutic agents), stimuli-responsive drug release (tumor microenvironment triggers such as pH, ATP, and glutathione can be incorporated for controlled release), and surface modification (effortless and smooth functionalization with polymers, ligands or imaging probes) [102,108,109].

Despite these advantages, porphyrin-based MOFs face a number of limitations that can hinder their biomedical application. The presence of metal ions or clusters may raise concerns regarding metal-related toxicity, particularly if degradation products accumulate or are released under physiological conditions. Furthermore, the stability of MOFs in body fluids can vary depending on the coordination chemistry of the metal–ligand system, potentially leading to premature degradation or alterations in the kinetics of porphyrin release [110]. Their highly porous and complex structure can also make it difficult to reproducibly control parameters such as particle size, porosity, drug loading, and surface properties during large-scale production. These limitations stem from the fact that the stability and biological behavior of MOFs are closely related to their composition, coordination structure, and physicochemical properties, highlighting the need for careful material selection, evaluation of degradation processes, and the use of standardized manufacturing procedures [111].

### 5.2. COFs

COFs are a promising alternative to MOFs. For long-term therapeutic applications where metal toxicity presents concerns, they are crystalline porous materials that are constructed from organic monomers linked to strong covalent bonds. COFs combine the photochemical advantages of porphyrins together with the structural benefits of COFs [112,113,114,115].

Compared to MOFs, porphyrin-based COFs offer several benefits and have been proposed as alternatives that may reduce concerns related to metal-ion accumulation. However, their safety profiles require formulation-specific evidence; the absence of metal nodes alone does not establish overall biocompatibility. A comprehensive safety assessment must evaluate the nature of their degradation products, which typically consist of organic monomer precursors released via the hydrolysis of reversible linkages (such as imine or hydrazone bonds) in physiological fluids. The biological clearance of these materials is strongly dictated by their post-degradation fragment size. While low-molecular-weight monomers may undergo renal filtration, larger hydrophobic porphyrin fragments or intact, highly stable frameworks risk long-term tissue retention within the reticuloendothelial system, particularly the liver and spleen. This underscores a critical trade-off between maintaining structural stability for therapeutic efficacy and ensuring adequate biodegradability for safe excretion. Additional benefits include flexible surface modification (covalent functionalization enables precise tuning of surface properties), enhanced ROS diffusion (pore sizes 1–10 nm porous structures facilitate O_2_ transport and ROS diffusion, mitigating limitations induced by hypoxia) and high loading capacity (porphyrin loading up to 30–50 wt %). Although the porous architecture of COFs can enhance O_2_ diffusion in vitro or within model systems, this does not in itself confirm therapeutic relief of hypoxia in a tumor microenvironment [116,117,118,119,120,121].

Recent advances have shown remarkable therapeutic efficacy with porphyrin-based COFs. The study investigated a size-tunable porphyrin-based COF functionalized with hyaluronic acid (HA) as a platform for sequential drug delivery and combined chemotherapy and photodynamic therapy. The treatment was associated with remodeling of the tumor microenvironment, including a reduction in extracellular matrix components and collagen deposition, which contributed to a decrease in tumor solid stress. Upon exposure to 660 nm laser light, the porphyrin-based COF produced reactive oxygen species (ROS), resulting in oxidative damage to tumor cell membranes through lipid peroxidation [122]. Their versatile porous architecture also allows integration with immune checkpoint inhibitors, such as anti-PD-1 antibodies for photodynamic immunotherapy combinations [121,123].

Despite the absence of metal ions, porphyrin-based COFs (covalent–organic frameworks) are not without limitations. The issues of long-term biocompatibility and biodegradation require further study, as strong covalent bonds that ensure structural stability can simultaneously hinder the materials’ breakdown and clearance of materials from the body [124]. Furthermore, variations in particle size, surface charge, porosity, and crystallinity can influence cellular uptake, biodistribution, and therapeutic efficacy. The achievement of synthetic reproducibility and the precise control of these parameters pose a challenge, given that the formation of COF structures is highly dependent on reaction conditions, monomer structure, and network architecture. Consequently, the excellent chemical stability and structural modifiability of COFs can simultaneously raise questions about biodegradability, bodily clearance, and manufacturing reproducibility [125].

### 5.3. Electrospun Nanofibers

Electrospinning is an innovative technique for encapsulating porphyrins within polymeric nanofibers. This creates advanced materials with high surface-to-volume ratios, controlled drug release profiles, and localized delivery (therapeutic). Particular advantages are seen for topical applications and localized PDT, where sustained photosensitizer release is desirable [39,126,127,128,129].

Electrospun nanofibers of the core shell, with porphyrins encapsulated in the core and biocompatible polymers such as polyvinyl alcohol and gelatin in the shell, provide controlled release kinetics [130,131,132]. For example, a TPPF_16_[S-CH_2_-COOH] porphyrin encapsulated in gelatin/PVA core shell fibers showed initial burst release followed by sustained release for minimum of 9 days, with selective phototoxic effects against cervical cancer cells while sparing normal cells [133].

The use of electrospinning extends to antimicrobial PDT (aPDT). Porphyrin-loaded fibers incorporated into wound dressings or medical device coatings provided localized infection control. The use of biodegradable polymers in these systems can potentially eliminate the need for surgical removal, improving patient comfort as well as reducing healthcare costs [130,131,132].

However, electrospun nanofibers also have certain limitations. Their application relies heavily on localized delivery, which restricts their utility for deep-seated or disseminated tumors. Drug release can also be influenced by fiber diameter, polymer composition, porosity, and the distribution of porphyrins within the fibers, making it difficult to achieve precise and reproducible release profiles [133]. Furthermore, residual solvents or degradation products of the polymer matrix may raise concerns regarding local tissue compatibility. These limitations stem from the strong dependence of electrospun fiber properties on processing parameters, such as polymer concentration, solvent composition, applied voltage, and collection conditions. Consequently, achieving consistent fiber morphology, porphyrin content, and release characteristics remains a significant challenge for scaling up and clinical application [134].

### 5.4. Liposomal and Polymeric Nanoparticle Systems

In porphyrin delivery, liposomes represent one of the most clinically advanced nanocarrier platforms. Liposomal encapsulation results in enhanced porphyrin solubility, extended circulation half-life, and enabled passive tumor targeting via the EPR effect. Modification of the surface with PEGylation (polyethylene glycol) further improves the pharmacokinetic profiles and also reduces the clearance of the reticuloendothelial system [135,136,137,138,139].

Hyaluronic acid-grafted liposomes, which contain porphyrins, have demonstrated improved tumor targeting through CD44 receptor-mediated endocytosis, while pH-sensitive liposomes enable triggered release in the acidic tumor microenvironment [140,141,142,143]. In particular, liposomal formulations of benzoporphyrin derivatives (Visudyne) are clinically approved against age-related macular degeneration. Ongoing trials are also exploring their utility in pancreatic and other cancers [144,145,146,147].

Polymeric nanoparticles based on biodegradable polymers, such as PLGA and chitosan, offer additional advantages in terms of structural stability, controlled release, and flexible surface functionalization [148,149]. Due to their biocompatibility, biodegradability and capacity for targeted delivery, polysaccharide-based systems such as chitosan, hyaluronic acid, and cyclodextrin have attracted particular attention [150,151,152].

Despite their clinical and technological advantages, systems based on liposomes and polymeric nanoparticles also exhibit significant limitations. Liposomal formulations may face problems such as physical instability, drug leakage, or changes in particle properties during storage, while PEGylation can limit cellular uptake and contribute to the so-called ‘PEG dilemma’ [153]. Polymeric nanoparticles can vary in terms of size, porphyrin loading, degradation rate, and drug release kinetics, all of which may affect their biological efficacy. In the case of inorganic nanoparticles, including gold and magnetic systems, their translation into clinical practice may be further complicated by concerns regarding their long-term persistence in the body, tissue accumulation, clearance, and potential toxicity. These limitations stem from the complex interplay between nanoparticle composition, surface properties, size, degradation behavior, and interactions with biological systems [154]. Consequently, standardized physicochemical characterization, long-term safety assessment, and reproducible manufacturing processes remain crucial for the development of clinically reliable nanoparticle-based porphyrin formulations. Table 1 shows representative experimental evidence for porphyrin-based photosensitizer platforms.

Across the representative studies in Table 1, several consistent patterns emerge. Framework encapsulation (MOFs, COFs) reliably improves photosensitizer monodispersity and reduces ACQ relative to free-photosensitizer or nonfunctionalized carrier controls, and several platforms directly target tumor hypoxia through endogenous O_2_ generation (BSA-MnO_2_/Ce6@ZIF-8, PCN-224-CaO_2_-HA, DOX/ZnO_2_@Zr-Ce6/Pt/PEG), achieving tumor growth inhibition or cell kill rates exceeding 95% in hypoxia-adapted designs. Active targeting ligands (folate, HA/CD44, HER2 antibodies) consistently increase selective uptake in receptor-positive models relative to non-targeted or receptor-negative controls. A partial exception is worth noting. In the anti-HER2–PPa immunoconjugate system, antibody conjugation improved cellular selectivity but reduced intrinsic phototoxicity per mole relative to free PPa, illustrating that targeting strategies are not uniformly beneficial and can trade potency for selectivity. No directly contradictory quantitative outcomes (that is, two studies reporting opposite effects for comparable interventions) were identified in this evidence base, which probably reflects the heterogeneity of platforms and models rather than genuine convergence.

A significant limitation across the evidence base is the near absence of direct, matched-condition comparisons to clinically approved photosensitizers (e.g., Photofrin, verteporfin, and talaporfin). Most studies benchmark against free unformulated dye or an internal formulation variant, constraining conclusions about improvement over current standard of care. The verteporfin–liposome study is the only platform benchmarked against a photosensitizer already in clinical use. Reporting treatment conditions is also uneven: irradiation wavelength, fluence, and power density are specified for roughly half of the included studies, limiting cross-study comparison of dose–response relationships. Most quantitative efficacy data come from subcutaneous xenografts in immunodeficient mice. Only the porphyrin lipoprotein/αPD-1 study used an immunocompetent model capable of revealing systemic immune effects, and only Sor@GR-COF-366 reported both subcutaneous and orthotopic outcomes. Long-term biodistribution, degradation product clearance, and chronic toxicity, particularly for MOF platforms containing Zr, Mn, or Pt, remain unreported for the majority of these systems, representing the most consistent translational gap identified across the evidence base.

## 6. Therapeutic Applications

### 6.1. PDT

PDT remains the most extensively investigated therapeutic application of porphyrin-based agents. In clinical settings, the PDT protocol usually involves topical or systemic photosensitizer administration, followed by a drug-light interval that allows for selective tumor accumulation, and ending with localized light irradiation at the tumor site [168].

Porphyrin-based PDT has demonstrated efficacy in various malignancies, including skin, lung, esophageal, head and neck, bladder, and prostate cancers. In early-stage and superficial tumors, the minimally invasive nature offers particular advantages in morbidity and organ preservation. This benefit is best documented for basal cell carcinoma, where randomized controlled trials and meta-analyses comparing topical PDT with surgical excision consistently report lower complete response rates for PDT than for surgery but significantly better cosmetic outcomes and reduced morbidity, positioning PDT as a preferred option for superficial or low-risk lesions, cosmetically sensitive sites, or patients not suited to surgery, rather than a direct efficacy substitute for excision [24,31,169]. For other indications discussed in this review (esophageal, endobronchial, and bladder applications), comparable head-to-head efficacy data against surgical resection are limited, and PDT is more commonly positioned as a palliative, adjunctive, or organ-preserving alternative for patients who are poor surgical candidates rather than as a demonstrated equivalent to surgery.

**Figure 3:**
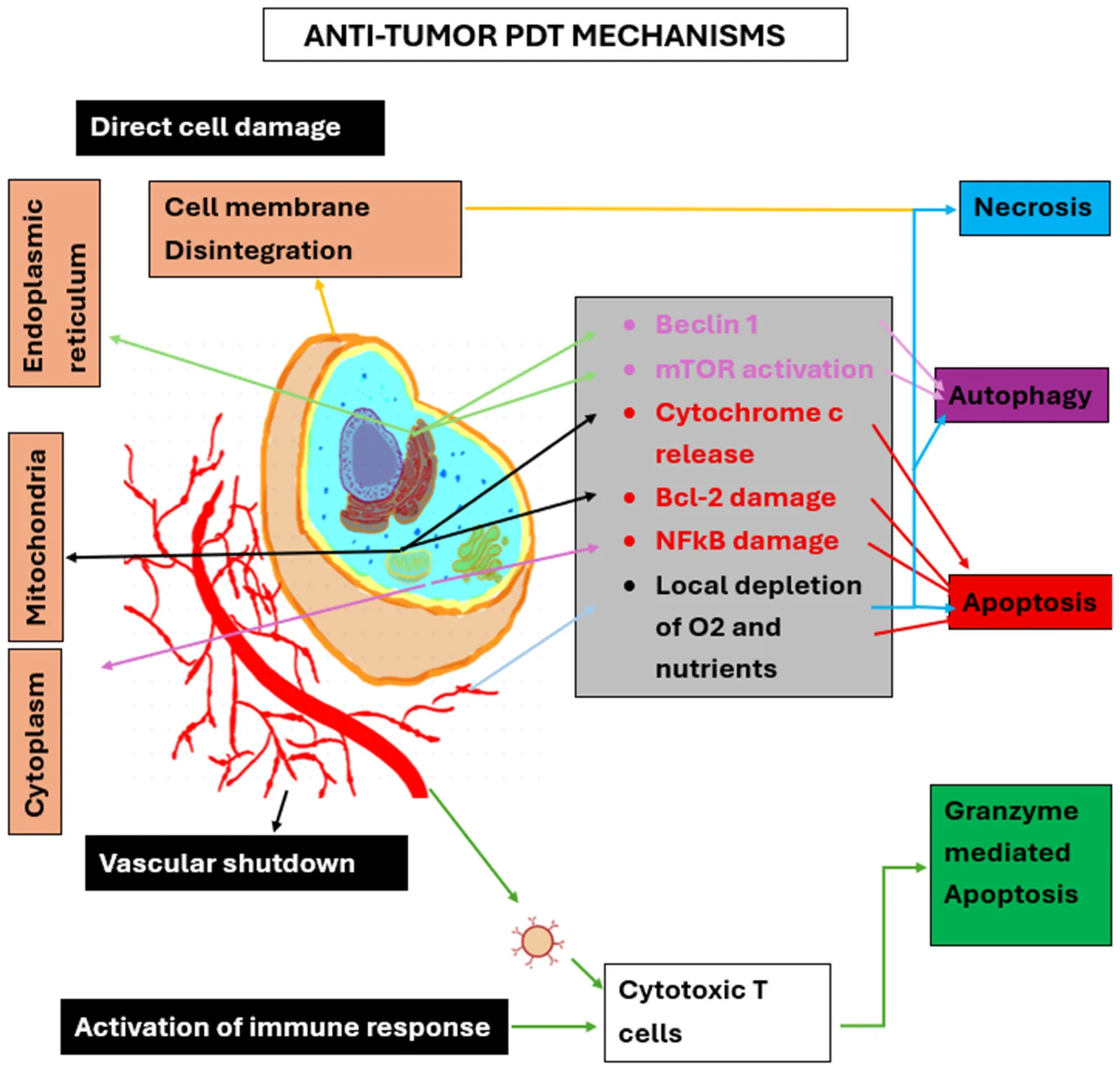
Tumor cell destruction via PDT. The schematic figure outlines the multifaceted antitumor photodynamic therapy (PDT) mechanisms, which collectively converge on tumor eradication through direct cellular damage, vascular disruption, and immune activation (black boxes). Direct cell damage occurs through oxidative damage to cell membrane and key organelles such as the endoplasmic reticulum, mitochondria, and cytoplasm (orange boxes) leading to intracellular dysfunction. Concurrently, cell membrane disintegration (orange box) is the result of cytoskeletal disruption and organellar damage, particularly affecting mitochondria and cytoplasmic structures. These trigger multiple cell death pathways, namely necrosis (blue), autophagy (purple), and apoptosis (red), the latter of which is further mediated by the release from cytotoxic T cells (green). At the molecular level, these processes are regulated by Beclin 1 (associated with autophagy), mTOR activation (modulating cell survival), mitochondrial cytochrome c release (initiating apoptosis), as well as damage to Bcl-2 and NFkB (key regulators of anti-apoptotic and inflammatory signaling). Beyond direct tumor cell killing, PDT also induces vascular shutdown, leading to local depletion of oxygen and nutrients, starving the tumor, while simultaneously activating a systemic immune response that enhances long-term antitumor immunity. Collectively, these interconnected pathways ensure comprehensive tumor suppression through combined cytotoxic, vascular, and immunological effects.

The therapeutic mechanisms of porphyrin-based PDT are multifaceted. Direct destruction/killing of tumor cells occurs through ROS-induced apoptosis, necrosis, or autophagy (Figure 3). In addition, PDT triggers vascular damage through endothelial cell injury and platelet aggregation. This leads to ischemia and tumor tissue hypoxia. The inflammatory response after PDT activates immune surveillance with DAMPs (damage-associated molecular patterns) released from dying tumor cells that promote dendritic cell maturation and T-cell activation. This phenomenon is termed “immunogenic cell death” [51].

The escalating challenge of antimicrobial resistance has increased interest in aPDT as a non-selective, mechanism-independent approach to infection control. Porphyrin-mediated aPDT has shown efficacy against bacteria (including multidrug resistant strains), fungi, viruses, and biofilms. The multiple targets of ROS, such as proteins, lipids, and nucleic acids, make resistance development highly unlikely, therefore positioning aPDT as a promising strategy to treat persistent infections [170,171,172].

### 6.2. Photothermal Therapy (PTT)

Although porphyrins are usually studied for their ROS-generating capabilities, certain derivatives and metalloporphyrins exhibit efficient photothermal conversion (the capacity to absorb light and dissipate energy as heat). Near-infrared-responsive porphyrin-based agents can induce localized hyperthermia (exceeding 45 °C), which is sufficient to denature proteins and induce coagulative necrosis. Through the integration of PDT and PTT within a single porphyrin-based nanoplatform, they offer synergistic therapeutic advantages. The hyperthermic conditions of PTT enhance photosensitizer uptake, increase tumor oxygenation, and sensitize cells to oxidative damage, while the ROS-mediated vascular disruption suppresses heat dissipation. This creates a positive feedback loop that amplifies therapeutic efficacy [173,174,175]. Importantly, formal synergy analysis using the α-value method (where α = (SFa × SFb)/SFab) has been performed using the value method (where = (SFa SFb)/SFab) for PDT-PTT combinations, with α values >1 confirming synergistic effects at specific light fluences [176].

### 6.3. Sonodynamic Therapy (SDT)

Sonodynamic therapy represents an innovative adaptation of the photodynamic principle. It substitutes ultrasound for light to activate sonosensitizers. Since ultrasound offers substantially deeper penetration of the tissue than light, it makes SDT particularly attractive for treating deep-located tumors that are inaccessible to conventional PDT. Porphyrin-based compounds exhibit sonsosentizing properties, and with nanotechnological encapsulation, their acoustic responsiveness is enhanced [177,178,179].

### 6.4. Synergistic Combination Therapies

The multifunctional capacity of porphyrin-based nanoplatforms facilitates sophisticated combination strategies that leverage complementary therapeutic mechanisms:

#### 6.4.1. Chemotherapy-PDT

By co-loading chemotherapeutic agents within porphyrin-based nanocarriers, it enables combined cytotoxic and photodynamic effects. The tumor microenvironment can be exploited for sequential drug release, with acidic pH and elevated levels of glutathione levels triggering chemotherapeutic release, while light-activated PDT generates localized ROS. Synergistic interactions between chemotherapeutics and ROS can potentially overcome multidrug resistance and reduce the required drug doses, thereby limiting systemic toxicity [180]. Formal synergy analysis has been performed for the chemo-PDT combinations. In a study using H1299 non-small cell lung cancer cells, Crescenzi et al. demonstrated that PDT combined with low-dose cisplatin or gemcitabine yielded therapeutic effects ranging from additive to clearly synergistic, with the most effective combination being PDT plus cisplatin. The synergy was attributed to disjointed cell cycle phase-related activity: PDT targeted G0-G1 cells and caused accumulation in the S phase, while low-dose cisplatin killed cells in the S phase, resulting in more pronounced therapeutic reinforcement. This synergistic interaction allowed for a dose reduction in the cytostatic drugs without compromising therapeutic efficacy [181].

#### 6.4.2. Immunotherapy-PDT

Immunogenic cell death induced by PDT can also be exploited to enhance the efficacy of the immune checkpoint inhibitor. As discussed earlier, porphyrin-based COFs loaded with anti-PD-1 antibodies have demonstrated remarkable tumor inhibition through combined photodynamic and immunotherapeutic mechanisms. In addition, PDT-induced release of tumor antigens can promote dendritic cell maturation (antigen-presenting cells) and T-cell priming, establishing systemic antitumor immunity [182,183,184].

#### 6.4.3. PDT-CDT Synergy

The combination of PDT with chemodynamic therapy (CDT) generates cytotoxic ROS through Fenton or Fenton-like reactions, which addresses the oxygen dependence of PDT. CTD can generate hydroxyl radicals even in hypoxic tumor environments, while PDT-generated ROS can potentiate CDT by releasing metal ions from Fenton catalysts. As such, metal-based porphyrin nanoparticles have been developed to take advantage of this combined PDT/CDT approach for enhanced efficacy against hypoxic tumors [185]. Although these studies describe enhanced combined effects for PDT-CDT platforms, formal synergistic analysis (e.g. Chou–Talalay combination index) does not appear to have been routinely reported, and further confirmation may be needed to substantiate synergistic claims.

## 7. Imaging Modalities and Theranostic Integration

The theranostic paradigm integrates diagnostic imaging capabilities within therapeutic platforms, enabling real-time treatment monitoring and personalized optimization. It is crucial to distinguish between the various signals that porphyrin-based theranostic platforms can report, since these do not necessarily convey the same information. Imaging of carrier or nanoplatform biodistribution, for example, a fluorescence, photoacoustic, or radionuclide signal from the intact porphyrin-MOF, liposome, or antibody conjugate indicates where the delivery vehicle has accumulated. However, it does not confirm by itself that the photosensitizer has been released, activated, or retains its photochemical activity at that site [186,187,188]. This distinction becomes critical for ‘always-on’ porphyrin formulations, where the intact and quenched molecule can still fluoresce weakly from the aggregated or bound states, potentially overestimating the pool of photodynamically active material [62]. Activatable photosensitizers address such a limitation more directly. Since their fluorescence and singlet-oxygen-generating capacity are switched on only upon a specific triggering event (such as protease cleavage, pH shift, or self-immolative linker degradation), the imaging signal itself reports the activation state of the drug rather than merely its presence [14,189]. Importantly, even accurate imaging does not guarantee that it predicts the therapeutic outcome. Pharmacokinetic studies with clinically used photosensitizers, such as temoporfin (Foscan), have shown a poor correlation between tissue photosensitizer concentration and PDT efficacy, with the treatment response instead more closely related to plasma photosensitizer levels and vascular effects [190]. This dissociation shows that carrier distribution, drug activation, and therapeutic response are mechanistically distinct endpoints that cannot be assumed to be interchangeable. Encouragingly, real-time theranostic reporters capable of directly signaling cell death or oxidative damage during treatment, rather than drug distribution, have been shown to enable dynamic imaging-guided dose optimization with a demonstrable improvement in the outcome of PDT. Therefore, it represents a more direct link between imaging readout and clinical benefit [96]. The broadest validation of this link across porphyrin-based nanoplatforms, particularly through prospective clinical studies correlating quantitative imaging biomarkers with treatment response, nevertheless remains an important unmet need for the field.

Porphyrin-based compounds are uniquely suited to this role, with intrinsic fluorescence and the capacity to accommodate various contrast agents.

### 7.1. Fluorescence Imaging

Porphyrin fluorescence provides imaging modality for tumor detection, surgical guidance, and also treatment monitoring. Emission in the near-infrared window (650–750 nm) enables deep tissue penetration with reduced autofluorescence interference. Fluorescence-guided surgery using porphyrin-based agents has been demonstrated for various tumors, including glioblastoma, lung, and ovarian cancers. This allows real-time visualization of tumor margins, as well as improving completeness of resection [185].

### 7.2. Photoacoustic Imaging

Photoacoustic imaging is a technique which combines optical excitation with acoustic detection, overcoming the limited penetration of purely optical based techniques. As discussed earlier, porphyrin-based compounds serve as an effective photoacoustic contrast agent, with absorption in the near infrared enabling deep tissue imaging with high spatial resolution. The same molecular properties that allow ROS generation for PDT can be used for photoacoustic imaging and can provide a direct means of visualizing photosensitizer distribution and predicting therapeutic efficacy [186,187].

### 7.3. MRI and PET/SPECT Imaging

In addition to fluorescence and photoacoustic imaging, the metal chelating capacity of porphyrins enables the integration of clinically established imaging modalities. Paramagnetic metal ions, such as gadolinium and manganese coordinated within porphyrin macrocycles, provide T_1_-weighted MRI contrast. However, radionuclide-labeled porphyrins, such as ^64^Cu, ^68^Ga, ^89^Zr, ^99m^Tc(acac)–TCPP complex, and ^111^In, enable PET or SPECT imaging. Porphyrin-based MOFs containing multiple metal ions can achieve multimodal imaging within a single platform and also provide complementary anatomical and functional information [188].

The incorporation of imaging capabilities within porphyrin-based theranostic platforms offers several clinical benefits, including accurate tumor staging and patient selection, real-time monitoring of the drug distribution and accumulation, evaluation of therapeutic response, and early detection of tumor recurrence.

## 8. Clinical Translation and Regulatory Considerations

### 8.1. Marketed Formulations

Several porphyrin-based photosensitizers have achieved formal regulatory approval, though approval status, exact indication, and jurisdiction differ substantially between agents. Porfimer sodium (Photofrin^®^) was first approved by the US FDA in 1995 for palliation of obstructive esophageal cancer, with subsequent approvals in 1998 for microinvasive endobronchial non-small cell lung cancer and in 2003 for ablation of high-grade dysplasia in Barrett’s. Verteporfin (Visudyne^®^) was approved by the FDA in April 2000 for predominantly classic subfoveal choroidal neovascularization due to age-related macular degeneration, with the indication extended in 2001 to pathologic myopia and presumed ocular histoplasmosis. It holds the corresponding approvals from regulatory agencies in approximately 30 other countries. 5-Aminolevulinic acid (Levulan^®^ Kerastick^®^) was approved by the FDA in December 1999 for nonhyperkeratotic actinic keratosis of the face and scalp, with approval later extended to Korea, Mexico, Brazil, Argentina, Chile and Colombia; methyl aminolevulinate (Metvix^®^/Metvixia^®^) received European approval (and formerly FDA approval, withdrawn from the US market in 2015) for actinic keratosis and superficial/nodular basal cell carcinoma, while hexaminolevulinate (Hexvix^®^/Cysview^®^) is approved for fluorescence-guided cystoscopic detection of non-muscle-invasive bladder cancer rather than as a therapeutic agent. Talaporfin sodium (Laserphyrin^®^) is approved in Japan, not internationally, for centrally located early-stage lung cancer (approved 2003–2004) and, since 2013, for primary malignant brain tumors under Japan’s National Health Insurance system. Temoporfin (Foscan^®^) was approved by the EMA in 2001 for the palliative treatment of advanced head and neck squamous cell carcinoma in patients who have failed previous therapies. Its pivotal trials showed improvement in a minority of patients rather than tumor eradication, and it is not FDA approved. Padeliporfin (TOOKAD^®^ Soluble) received EMA approval in 2017 for vascular-targeted photodynamic therapy of low-risk localized prostate cancer. It remains investigational in the United States, where it holds Fast Track and Orphan Drug designations but has not received FDA approval. Several additional agents are referenced in Table 1, including tin ethyl etiopurpurin, motexafin lutetium, Photosens, and Radachlorin, which reached clinical trials or regional approval (Russia, CIS countries) but did not have broad international regulatory authorization or were discontinued. These are presented for historical and comparative context rather than as current standard-of-care options [144,145,146,147,191,192].

### 8.2. Clinical Challenges and Barriers

Despite progress, significant barriers impede broader clinical translation of porphyrin-based theranostics.

#### 8.2.1. Phototoxicity and Cutaneous Photosensitivity

Prolonged photosensitivity followed by systemic administration remains a major concern and requires patients to avoid light exposure for extended periods, hence limiting the patient’s quality of life and therapeutic convenience. It should be noted that third-generation agents with rapid clearance and tumor-specific activation aim to address this limitation [193,194].

#### 8.2.2. Tissue Penetration Limitations

Due to visible and near-infrared absorption, most of them restrict effective PDT to lesions within approximately 2 cm of tissue surfaces. But the near-infrared-absorbing derivative and sonodynamic approaches offer improved penetration. However, deep-located tumors remain challenging [193,195].

#### 8.2.3. Hypoxia

As a result of the oxygen dependency of Type II ROS generation, it renders PDT less effective in hypoxic tumor environments. Such environments are common in advanced malignancies. Combination therapies that integrate chemotherapy or CDT with porphyrin-based compounds can partially overcome this limitation. However, strategies for independent mechanisms for tumor oxygenation or hypoxia are needed [193,195,196,197].

#### 8.2.4. Tumor Heterogeneity

Variable uptake, sensitivity, and distribution between different tumors and also within individual tumors complicate the standardization of treatment and prediction of response [198].

#### 8.2.5. Biosafety and Long-Term Toxicity

The biodistribution, clearance, and chronic toxicity of porphyrin-based nanomaterials often require a comprehensive evaluation. In addition, for MOF-based systems, concerns regarding metal-ion accumulation and potential long-term toxicity necessitate the need for careful material design and rigorous safety assessment [199].

#### 8.2.6. Manufacturing and Regulatory Hurdles

The complexity of multifunctional nanoplatforms poses huge challenges for reproducible manufacturing, quality control, and regulatory approval. Standardized characterization methods and stability protocols are required to ensure batch-to-batch consistency and clinical reliability [200].

## 9. Future Perspectives

The future of porphyrin-based medicine lies in addressing the current limitations through innovative design strategies and expanding therapeutic applications.

### 9.1. Precision Molecular Engineering

Advanced synthetic approaches can enable precise tuning of porphyrin properties including absorption wavelength, ROS quantum yield, and pharmacokinetic behavior. Computational modeling and high-throughput screening will accelerate the identification of optimal structures for specific clinical applications [201].

### 9.2. Near-Infrared-Activated Agents

The development of porphyrin-based agents with absorption in the second near-infrared window (100–1700 nm) will dramatically improve tissue penetration, hence enabling treatment for deep-seated tumors. Strategies such as porphyrin dimerization, π-extension, and heavy atom substitution are being explored to achieve this objective [202,203].

### 9.3. Responsive and ‘Smart’ Systems

Tumor microenvironment-responsive nanoplatforms that are activated by low Ph, elevated glutathione, hypoxia, or specific enzymes will enable targeted delivery and activation at the tumor site and minimize off-target effects. Such ‘smart’ systems can substantially reduce phototoxicity and improve therapeutic indicators [204,205,206].

### 9.4. Immunomodulatory Combinations

Since it is recognized that PDT induces immunogenic cell death, it opens opportunities for combination with immune checkpoint inhibitors, adaptive cell therapy, and cancer vaccines. Porphyrin-based platforms, which co-deliver immunomodulatory agents, can potentially establish durable immunity and prevent recurrence [207].

### 9.5. Personalized Theranostics

The integration of various capabilities such as diagnostic, therapeutic, and monitoring within a single platform enables personalized treatment optimization. Real-time imaging-guided PDT and feedback-controlled dosing will allow individualized therapy based on patient-specific tumor characteristics and treatment response [95].

### 9.6. Expanded Therapeutic Indications

Beyond oncology, porphyrin-based compounds are promising for applications including cardiovascular disease (atherosclerosis), dermatological conditions, infectious disease, and neurological disorders, where localized oxidative stress could be beneficial [171,198,208,209]. Table 2 shows a comparison of first- and second-generation porphyrins.

Table 3 shows a comparison of third-generation photosensitizer delivery systems.

**Figure 4:**
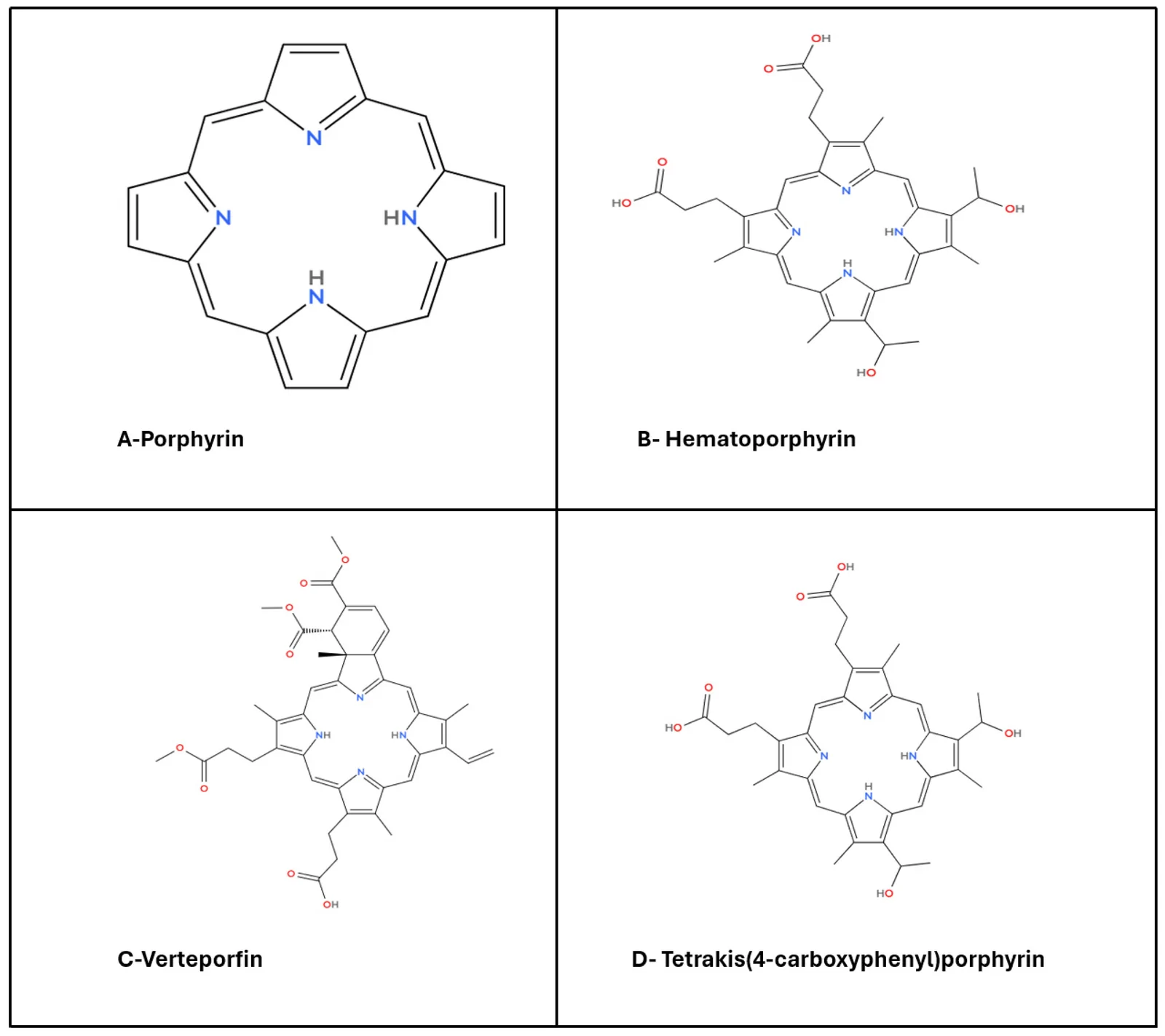
Structural evolution of porphyrin-based photosensitizers between generations (A → B → C → D). (**A**) Porphyrin, the unsubstituted parent tetrapyrrole macrocycle common to all porphyrin derivatives, consisting of four pyrrole subunits linked by four methine bridges; this core scaffold underlies naturally occurring tetrapyrroles such as heme and chlorophyll. (**B**) Hematoporphyrin, a first-generation photosensitizer that contains hydroxyethyl and propanoic acid substituents in the porphine core; its purified derivative (hematoporphyrin derivative, HpD) formed the basis of sodium porfimer (Photofrin^®^), the first clinically approved PDT agent (Table 2). (**C**) Verteporfin (Visudyne^®^), a second-generation benzoporphyrin derivative with a reduced ring-fused structure; it is FDA-approved for photodynamic treatment of neovascular age-related macular degeneration and is the photosensitizer used in the verteporfin-loaded liposome platform described in Table 1 (**D**) Tetrakis(4-carboxyphenyl)porphyrin (TCPP), a third-generation porphyrin that contains four peripheral carboxyphenyl groups that enable coordination with metal clusters; it serves as an organic ligand in the porphyrinic metal-organic framework, discussed as a representative nanoplatform in Section 5.1. The progression from A to D illustrates increasing structural complexity and functionalization accompanying the transition from early hematoporphyrin derivatives to nanoplatform-integrated photosensitizers.

Figure 4 shows the structural evolution of porphyrin-based photosensitizers.

## 10. Conclusions

Porphyrin-based compounds have evolved from the conceptual origins of PDT to the sophisticated frontiers of theranostic nanomedicine. Their unique combination of factors, such as tumor selectivity, tunable photophysical properties, and intrinsic imaging capabilities, have established them as one of the most adaptable platforms in contemporary biomedical research. Their evolution from first-generation hematoporphyrin derivatives to third-generation nanotheranostic systems has exemplified the power of interdisciplinary collaboration. Therefore, we synthesize advances in organic chemistry, nanotechnology, materials science, and molecular biology to address the unfavorable persistent challenges of cancer management.

The fundamental limitations of porphyrins, such as solubility, stability, and targeting, can be examined by integrating porphyrins with nanocarrier systems, particularly MOFs, COFs, and electrospun fibers, while enabling sophisticated multifunctionality. The capacity to simultaneously image, treat, and therapeutic monitoring together or within a single platform represents a favorable shift toward personalized and precision oncology. As the challenges of clinical translation including phototoxicity, tissue penetration, hypoxia, and regulatory approval are progressively addressed, porphyrin-based chemicals are prepared to make significant contributions to cancer treatment. It aims to improve the outcome while minimizing the toxicity and morbidity associated with conventional therapies.

However, to advance, it requires continued fundamental research into porphyrin photochemistry and tumor biology; technological innovation in nanoplatform design and manufacturing; preclinical and clinical evaluation; and sustained collaboration among academic researchers, clinicians, and industry partners. With these collaborative efforts, the remarkable potential of porphyrin-based compounds can be utilized from the laboratory to the clinic, fulfilling their promise as both diagnostic probes and therapeutic agents in the fight against cancer.

## Figures and Tables

**Table 1:** Representative experimental evidence for porphyrin-based photosensitizer platforms.

Platform/Porphyrin Design	Biological Model & Experimental Groups	Quantitative Results & Primary Outcomes	Critical Controls & Benchmark Comparisons	Demonstrated Added Value	Key Limitations & Translational Gaps	References
Sor@GR-COF-366	HepG2/Huh7 cells; subcutaneous and orthotopic HCC mouse models.	~97% TGI in subcutaneous tumors and ~98% TGI in orthotopic tumors; prolonged survival reported.	Untreated/PBS and formulation controls.	GalNAc-mediated ASGPR targeting plus co-delivery of sorafenib produced strong antitumor activity.	Multicomponent system; Long-term COF degradation, biodistribution and manufacturing require further evaluation.	[155]
Cationic TAPP–VIO COF	*S. aureus* and *E. coli*; infected-wound mouse model.	^1^O_2_ generation ~179% higher than non-ionic porphyrin COF; colonies reduced to ~0.81% (*S. aureus*) and ~1.16% (*E. coli*) under tested conditions.	Non-ionic porphyrin COF and untreated controls; 100 mW/cm^2^ irradiation.	The cationic surface enhances electrostatic interaction with negatively charged bacterial envelopes.	Systemic safety, long-term biodegradation, and translation beyond wound infection remain uncertain.	[156]
BSA–MnO_2_/Ce6@ZIF-8	Cancer cell and mouse tumor models reported in the original study.	Ce6 loading ~28.3 wt%; enhanced PDT under 650 nm, 230 mW/cm^2^ irradiation; MnO_2_ generates O_2_ and Mn^2+^ provides MRI contrast.	Free/alternative Ce6 and carrier controls.	O_2_ generation addresses hypoxia, while ZIF-8 improves hydrophobic Ce6 delivery and imaging.	Long-term serum stability, Mn/Zn clearance, degradation products, and systemic toxicity require study.	[157]
PCN-224–CaO_2_–HA	4T1, MCF-7, and LO2 cells; 4T1 tumor-bearing mice.	Post-PDT cell survival ~14.58% in 4T1 and ~22.45% in MCF-7; enhanced tumor inhibition in vivo.	Free/loaded TCPP and carrier controls; targeting/O_2_-generation controls.	HA/CD44 targeting plus CaO_2_-mediated O_2_ generation improves PDT under hypoxia.	CaO_2_ reactivity, MOF persistence, and long-term systemic safety need clarification.	[158]
Localized PVA–gelatin/porphyrin core–shell nanofibers	HeLa cervical cancer cells and nontumor comparator cells; in vitro localized model.	Singlet oxygen quantum yield ΦΔ ≈ 0.62; sustained release 9 days; greater phototoxicity toward tumor cells than non-tumor cells.	Nanofiber without PS and dark controls; tumor/non-tumor comparison.	Localized sustained delivery may reduce systemic exposure and improve treatment in accessible lesions.	In vitro only; implantability, biodegradation, inflammation, and light penetration need in vivo validation.	[159]
Verteporfin-loaded lipid–polymer liposome	T98G glioblastoma cells.	~98% reduction in T98G viability at 1.0 μmol/L verteporfin with 470 nm activation.	Free verteporfin and unloaded liposome controls.	The liposome improves the formulation of verteporfin and intracellular delivery while allowing fluorescence-based localization.	In vitro only; 470-nm activation has limited tissue penetration for deep tumors.	[160]
ZIF-8@Ce6–HA	HepG2 hepatocellular carcinoma cells.	~88.4% HepG2 cell death after PDT; HA formulation increased circulation time and reduced systemic toxicity	Unmodified ZIF-8@Ce6 and dark/vehicle controls.	HA-mediated targeting of CD44 adds active localization to MOF-based Ce6 delivery.	In vitro evidence; CD44 heterogeneity and in vivo targeting need confirmation.	[161]
PEGylated TCPP dual-stimuli micellar nanoparticles	4T1 triple negative breast cancer cells and tumor-bearing mice, including lung metastasis model.	Suppressed primary tumor growth and lung metastases under combined PDT/chemotherapy; fluorescence capability.	Drug-free/nonactivated formulations and treatment controls.	Dual pH/MMP-2 activation improves tumor selective release and combines PDT with DOX chemotherapy.	Exact cross-study quantitative comparisons are limited; pharmacokinetics and long-term fate remain gaps.	[162]
Folate-modified PLGA–Pba nanoparticles	MKN28 gastric cancer cells; MKN28 tumor-bearing mice.	~200 nm particles; faster uptake than nontargeted PLGA-Pba; high tumor accumulation within 24 h and enhanced tumor killing.	Free Pba and nontargeted PLGA-Pba controls.	Folate/FR targeting improves tumor localization and exposure relative to free and untargeted Pba.	The magnitude of receptor-specific delivery versus passive accumulation is not fully resolved; Long-term biodistribution needs study.	[163]
Anti-HER2–PPa immunoconjugates	HER2+ SK-BR-3/SK-OV-3 and HER2 MDA-MB-468 cells.	PPa: mAb ratios up to ~10; selective phototoxicity toward HER2+ cells after 670 nm irradiation; Multiepitope targeting improved efficacy versus single-epitope targeting.	Free PPa and HER2 cells.	Improves cellular selectivity, but conjugation can reduce intrinsic phototoxicity per mole versus free PPa.	No in vivo validation; HER2 heterogeneity and antibody penetration are translational limitations.	[164]
Porphyrin lipoprotein + αPD-1	AE17-OVA dual subcutaneous tumor model in C57BL/6 mice.	IL-6 increased ~46.64-fold after repeated PDT and ~61.33-fold after PDT + αPD-1; increased responses of MHC II/CD80/CD86 and distal CD8+ responses; abscopal effects reported.	PBS, repeated PDT, αPD-1 and combination groups.	Demonstrates systemic immune effects beyond local phototoxicity and enhanced response with checkpoint blockade.	Preclinical immunotherapy model; Inflammatory toxicity, optimal dosing and human translation remain uncertain.	[165]
DOX&ZnO_2_@Zr-Ce6-Pt/PEG	Tumor-bearing mice and cancer cell models reported in the original study.	~2.94-fold enhancement of ^1^O_2_ under acidic conditions; ~96.8% TGI reported; intratumoral 630 nm irradiation.	Free/partial formulations and untreated controls.	Endogenous generation of H_2_O_2_/O_2_, pH-responsive release, nanozyme activity, and combination enhance treatment.	Intratumoral administration limits generalizability; long-term Zr/Pt/Zn clearance and DOX-associated toxicity remain concerns.	[166]
AuNPs@TMPyP	MDA-MB-231 TNBC cells, healthy breast cells, 2D/3D spheroids.	Red laser PDT (40 J/cm^2^) combined with X-rays or proton therapy increased ROS, reduced survival fraction, loss of mitochondrial membrane potential, and promoted apoptosis; proton therapy was more effective than X-rays.	AuNPs, TMPyP alone, laser alone, X-rays/protons alone and untreated controls.	Multimodal radiation + PDT increases tumor cell damage while retaining in-vitro selectivity.	In vitro only; long-term gold fate, biodegradation, scalability, and in vivo efficacy remain unresolved.	[167]

**Table 2:** Comparative table of first-generation and second-generation porphyrins.

Drug	Generation	Trade Name	Chemical Class	Clinical Status	Indication	Target Mechanism	Activation Wavelength	Major Advantages	Major Limitations	Improvements Achieved by Later Generations	Jurisdiction	Regulatory Source
Hematoporphyrin	First	-	Natural porphyrin mixture derived from heme metabolism	Historical; no longer used as a primary therapeutic agent	Early tumor fluorescence detection; experimental PDT studies in solid tumors	Passive tumor accumulation through altered vasculature, increased permeability, and binding to cellular components	~630 nm	First demonstration of preferential tumor accumulation and phototoxicity	Poor chemical purity; mixed porphyrin species; low tumor selectivity; weak predictability	Purified derivatives with improved composition and pharmacokinetics	Not applicable; historical compound	-
Hematoporphyrin derivative	First	Photofrin I (historical designation)	Partially purified HpD containing monomers, dimers, and oligomers	Historical; early clinical PDT trials	Glioma, bladder, lung, esophageal, and skin tumors in early trials	Lipoprotein binding, vascular retention, preferential malignant tissue uptake, and ROS generation	~630 nm	Improved tumor localization; established clinical PDT concept	Complex mixture; variable composition; prolonged photosensitivity; limited penetration	Led to porfimer sodium and later chlorins/bacteriochlorins	Not applicable; historical investigational preparation	-
Porfimer sodium	First	Photofrin^®^	Purified hematoporphyrin derivative containing oligomeric porphyrins	Approved drug; used in selected countries	Esophageal cancer, NSCLC, bladder cancer, cholangiocarcinoma, Barrett’s esophagus, and other region-dependent indications	Tumor/tumor-vascular accumulation; photoinduced singlet oxygen and other ROS causing apoptosis, necrosis, and vascular damage	630 nm	First internationally approved PDT photosensitizer; extensive evidence; useful for obstructing and superficial tumors	Prolonged skin photosensitivity; low penetration; heterogeneous composition; delayed clearance	Later agents improved wavelength, purity, selectivity, and clearance	USA (FDA approved), approved in Canada and Japan	FDA approval: esophageal cancer (1995), NSCLC (1998), Barrett’s esophagus HGD (2003)
5-Aminolevulinic acid	Second	Levulan^®^, Gliolan^®^	Porphyrin precursor/prodrug converted to PpIX	Approved; clinical use	Actinic keratosis, fluorescence-guided glioma surgery, and dermatological PDT; indications vary by product	Transformation of heme biosynthesis to mitochondrial PpIX, followed by ROS generation	~635 nm	Metabolic selectivity; short photosensitivity; diagnostic fluorescence	Variable PpIX production; metabolic dependence; limited deep-tissue penetration	Introduced tumor-selective metabolic activation	Levulan^®^-USA (FDA), EU, Korea, Mexico, Brazil, Argentina, Chile, Colombia. Gliolan^®^-EU	Levulan^®^-FDA approval, December 1999- actinic keratosis of the face/scalp, EMA Gliolan EPAR. Gliolan^®^-EMA APPROVAL (fluorescence-guided resection of malignant glioma)
Methyl aminolevulinate	Second	Metvix^®^	ALA methyl ester	Nationally authorized in specified countries	Actinic keratosis; superficial and nodular basal cell carcinoma	Cellular conversion to PpIX and mitochondrial ROS generation	~630–635 nm	Greater lipophilicity and skin penetration than ALA	Primarily superficial use; variable response in thick lesions	Improved lipophilicity and tissue penetration	EU (EMA); formerly USA (FDA, withdrawn from US market 2015)	EMA marketing authorization-actinic keratosis, superficial/nodular BCC
Hexaminolevulinate	Second	Hexvix^®^, Cysview^®^	ALA hexyl ester	Approved for diagnostic use	Fluorescence-guided detection of non-muscle-invasive bladder cancer	Enhanced urothelial uptake and PpIX formation	~635 nm	Improved tumor visualization over white-light cystoscopy	Diagnostic rather than systemic therapeutic use; inflammatory false positives	Improved cellular uptake compared with ALA	USA (FDA) as Cysview; European Union (EMA) as Hexvix	FDA/EMA approval fluorescence cystoscopy for nonmuscle-invasive bladder cancer detection (diagnostic, not therapeutic)
Verteporfin	Second	Visudyne^®^	Benzoporphyrin derivative monoacid ring A	Approved	Neovascular age-related macular degeneration and choroidal neovascularization	LDL-associated endothelial localization; vascular PDT causes endothelial injury and thrombosis	689 nm	Longer activation wavelength; rapid clearance; established ophthalmic use	Limited cancer application; injection-related adverse effects; specialized delivery	Improved penetration and clearance compared with Photofrin	USA (FDA); also EMA and ~30 additional countries	FDA approval, April 2000 (AMD); extended 2001 to pathologic myopia and ocular histoplasmosis
Temoporfin	Second	Foscan^®^	Chlorin derivative: m-THPC	European Union-authorized	Palliative treatment of advanced head-and-neck squamous-cell carcinoma	Tumor-membrane, mitochondrial and lysosomal localization; high singlet-oxygen generation	652 nm	High photosensitizing efficiency; lower dose than Photofrin	Severe photosensitivity; narrow therapeutic window; formulation challenges	Greater potency and longer absorption wavelength than first-generation agents	EU	EMA marketing authorization, 24 October 2001-palliative treatment of advanced head and neck SCC
Talaporfin sodium	Second	Laserphyrin^®^	Chlorin e6 derivative	Approved in Japan	Early-stage lung cancer and primary malignant brain tumor indications in Japan	Cellular and membrane localization; mitochondrial and lysosomal ROS generation	664 nm	Faster clearance and shorter photosensitivity than Photofrin	Limited global availability; requires controlled light delivery	Reduced prolonged phototoxicity	Japan	PMDA approval early stage central lung cancer (2003–2004); malignant brain tumor (2013), under the Japanese National Health Insurance system
HPPH	Second	Photochlor^®^	Pyropheophorbide-a chlorin derivative	Investigational; clinical trials	Lung, esophageal, and head-and-neck cancers investigated	Lipoprotein-associated uptake and mitochondrial localization	665 nm	High extinction coefficient; potentially lower skin toxicity than Photofrin	Not commercially approved; investigational	Improved purity and pharmacokinetics over HpD	USA; investigational	No approval; Phase I/II clinical trials
Motexafin lutetium	Second	Lutex^®^	Texaphyrin metal complex	Investigational; development discontinued	Prostate cancer, breast cancer, and brain metastases investigated	Mitochondrial/membrane localization and oxidative damage	~730 nm	Very long wavelength; deeper penetration	Limited efficacy; discontinued development	Addressed penetration limitations of earlier porphyrins	USA; investigational, development discontinued	No approval; discontinued clinical trials
Padeliporfin	Second	WST11/TOOKAD soluble; Tookad^®^	Palladium bacteriopheophorbide	European Union-authorized	Vascular-targeted PDT for selected low-risk localized prostate cancer	Predominantly vascular localization; endothelial destruction and vascular occlusion	763 nm	Deep penetration; minimal prolonged skin photosensitivity	Requires intravenous administration and specialized light fibers	Shifted emphasis toward vascular targeting	EU (EMA) and Mexico approved; USA investigational only	EMA approval, 2017 vascular-targeted PDT for low-risk localized prostate cancer; US FDA Fast Track/Orphan Drug designation, not approved
Tin ethyl etiopurpurin	Second	Purlytin^®^	Purpurin derivative	Investigational; development discontinued	AMD and solid tumors investigated	Vascular and cellular ROS-mediated damage	~660 nm	Strong red absorption; singlet-oxygen generation	Limited clinical success; discontinued	Improved optical properties over porfimer sodium	USA; investigational, development discontinued	No approval; discontinued clinical trials
Photosens	Second	-	Sulfonated aluminum phthalocyanine mixture	Regional clinical use reported	Skin, esophageal, and other tumors reported	Membrane and intracellular ROS production	~675 nm	Strong absorption; high quantum yield	Limited international regulatory documentation	Improved absorption and photochemical efficiency	Russia; reported regional use	Approved by the Russian national regulatory authority; not approved elsewhere
Radachlorin	Second	-	Chlorin e6 derivative mixture	Regional clinical use reported	Skin cancer, cervical lesions, and superficial tumors reported	Cellular ROS generation after chlorin activation	~662 nm	Water solubility; favorable optical properties	Limited high-quality randomized evidence	Improved solubility compared with hydrophobic porphyrins	Russia/CIS; reported regional use	Approved for regional clinical use; no FDA/EMA approval
Photodithazine	Second	-	Chlorin e6 derivative	Clinical studies/regional use reported	Dermatology and superficial malignancies investigated	Intracellular ROS production	~660 nm	Improved solubility and photochemical activity	Limited international clinical validation	Improved formulation compared with earlier chlorins	Russia; reported regional use	No FDA/EMA approval; regional clinical studies only

This table provides a comparative overview of photosensitizing agents developed across multiple generations for photodynamic therapy (PDT), organized by drug name, generation, trade name, chemical class, clinical status, indications, target mechanism, activation wavelength, key advantages and limitations, improvements achieved by later generations, jurisdiction, and regulatory source.

**Table 3:** Comparative table of third-generation photosensitizer delivery systems.

System/Example	Targeting Strategy	Porphyrin/Photosensitizer Component	Clinical Status	Potential Disease Applications	Localization/Mechanism	Advantage over Second Generation	Remaining Limitations
Liposomal porphyrins (e.g., liposomal benzoporphyrin derivatives, liposomal chlorins)	Encapsulation in lipid bilayers improves delivery	Verteporfin, chlorins, porphyrin derivatives	Mainly preclinical; some formulations investigated clinically	Solid tumors, melanoma, pancreatic cancer, glioma	Liposomes accumulate through the enhanced permeability and retention (EPR) effect; intracellular release generates ROS	Improve solubility and reduces aggregation of hydrophobic porphyrins	Variable EPR effect in humans; limited active targeting
PEGylated porphyrins	Polyethylene glycol modification improves circulation time	Porphyrins, chlorins, bacteriochlorins	Preclinical	Solid tumors	Increased plasma half-life; passive tumor accumulation; ROS-mediated cell death	Reduces rapid clearance and improves pharmacokinetics	PEG shielding may reduce cellular uptake (“PEG dilemma”)
Porphyrin polymer nanoparticles	Polymer-based encapsulation or polymerized porphyrin structures	Porphyrins/chlorins incorporated into polymers	Preclinical	Cancer PDT, imaging-guided therapy	Nanoparticle Uptake Followed by intracellular ROS production	Improved stability, tumor accumulation, and multifunctionality	Manufacturing complexity; limited clinical translation
Porphyrin dendrimers	The branching macromolecular architecture controls delivery	Metalloporphyrins and chlorins	Preclinical	Solid tumors	Enhanced cellular uptake; ROS generation after irradiation	Higher loading capacity and tunable targeting	Difficult synthesis; toxicity evaluation required
Folate-targeted porphyrins	Folic acid ligand targets folate receptor-overexpressing tumors	Porphyrin/chlorin derivatives conjugated to folate	Preclinical	Ovarian cancer, breast cancer, lung cancer	Folate receptor-mediated endocytosis increases intracellular photosensitizer concentration	Improves tumor selectivity compared with passive accumulation	Folate receptor heterogeneity among tumors
Antibody–porphyrin conjugates	Monoclonal antibody targeting tumor antigens	Porphyrins linked to antibodies	Preclinical, early translational studies	HER2-positive breast cancer, EGFR-positive tumors, hematological malignancies	Antibody binding increases tumor-cell specificity; PDT causes ROS-mediated apoptosis	Highly selective targeting	The large molecular size limits penetration; expensive production
HER2-targeted porphyrin conjugates	HER2 receptor targeting	Porphyrin related to anti-HER2 antibodies/fragments	Preclinical	HER2-positive breast and gastric cancers	Receptor-mediated internalization and ROS production	Enables molecularly targeted PDT	Depends on HER2 expression; antibody limitations
Peptide-targeted porphyrins	Tumor-homing peptides (RGD, bombesin, other ligands)	Chlorins/porphyrins conjugated with peptides	Preclinical	Integrin-positive tumors, prostate cancer, breast cancer	Peptide receptor binding enhances tumor uptake	Smaller size improves penetration compared with antibodies	Stability and receptor specificity challenges
Transferrin-targeted porphyrins	Targets transferrin receptors overexpressed in tumors	Porphyrin-transferrin conjugates	Preclinical	Brain tumors, rapidly proliferating cancers	Receptor-mediated uptake followed by ROS production	Improves tumor-cell selectivity	Expression of the transferrin receptor expression in normal tissues
Gold nanoparticle–porphyrin systems	Gold nanoparticle carrier and photothermal/PDT combination	Porphyrins attached to gold nanoparticles	Preclinical	Solid tumors	Combined photodynamic and photothermal effects	Enables multimodal therapy and imaging	Long-term nanoparticle safety concerns
Silica nanoparticle–porphyrin systems	Mesoporous silica delivery	Porphyrins loaded into silica nanoparticles	Preclinical	Cancer PDT and imaging	Controlled release and enhanced intracellular delivery	Improves stability and prevents aggregation	Clearance and regulatory issues
Magnetic nanoparticle–porphyrin systems	Magnetic guidance and imaging capability	Porphyrins attached to iron oxide nanoparticles	Preclinical	Brain activated in acidic tumor, solid tumors	MRI-guided localization plus PDT	Combines diagnosis and therapy	Magnetic nanoparticle toxicity and clinical translation barriers
pH-responsive porphyrins	Activated in the acidic tumor microenvironment	Modified porphyrins	Preclinical	Solid tumors	Increased ROS generation after tumor-specific activation	Reduces normal tissue toxicity through selective activation	Complex Synthesis and validation
Enzyme-activatable porphyrins	Tumor enzyme activation (MMPs, proteases, etc.).	Activatable porphyrin derivatives	Preclinical	Cancer with elevated protease activity	‘Off-on’ activation improves tumor specificity	Reduces systemic toxicity	Requires reliable tumor biomarker expression
Hypoxia-responsive porphyrins	Tumor oxygen-sensitive activation	Modified porphyrins/bacteriochlorins	Preclinical	Hypoxic solid tumors	Designed to overcome PDT oxygen dependence	Addresses tumor hypoxia limitation	Complex biological validation
Porphyrin theranostic systems	Combined fluorescence imaging + PDT	Porphyrin-based multifunctional agents	Translational/Preclinical	Glioma surgery, cancer imaging-guided PDT	Intrinsic fluorescence allows localization and treatment guidance	Combines diagnosis and therapy in one molecule	Regulatory complexity

This table provides a systematic overview of third-generation porphyrin-based PDT delivery systems, categorized by the specific targeting strategy employed to overcome the limitations inherent in first- and second-generation photosensitizers.

## Data Availability

No new data were created or analyzed in this study. Data sharing is not applicable to this article.

## References

[1] Chen M., Ji S., Liu X., Zheng X., Zhou M., Wang W. (2025). Porphyrins and Their Derivatives in Cancer Therapy: Current Advances, Mechanistic Insights, and Prospective Directions. Mol. Pharm..

[2] Yang F., Xu M., Chen X., Luo Y. (2023). Spotlight on porphyrins: Classifications, mechanisms and medical applications. BioMed. Pharmacother..

[3] Lee H., Hong K.I., Jang W.D. (2018). Design and applications of molecular probes containing porphyrin derivatives. Coord. Chem. Rev..

[4] Ko Y.J., Lee M.E., Cho B.H., Kim M., Hyeon J.E., Han J.H., Han S.O. (2024). Bioproduction of porphyrins, phycobilins, and their proteins using microbial cell factories: Engineering, metabolic regulations, challenges, and perspectives. Crit. Rev. Biotechnol..

[5] Bonkovsky H.L., Guo J.T., Hou W., Li T., Narang T., Thapar M. (2013). Porphyrin and heme metabolism and the porphyrias. Compr. Physiol..

[6] Teixeira R., Serra V.V., Botequim D., Paulo P.M.R., Andrade S.M., Costa S.M.B. (2021). Fluorescence Spectroscopy of Porphyrins and Phthalocyanines: Some Insights into Supramolecular Self-Assembly, Microencapsulation, and Imaging Microscopy. Molecules.

[7] Kim B.F., Bohandy J. (1981). Spectroscopy of porphyrins. Johns Hopkins APL Tech. Dig..

[8] Phillips J.D. (2019). Heme biosynthesis and the porphyrias. Mol. Genet. Metab..

[9] National Library of Medicine (2025). Porphyrin Tests: MedlinePlus Medical Test.

[10] Testing.com (2020). Porphyrin Tests: What They Measure and How They Work. https://www.testing.com/tests/porphyrin-tests/.

[11] Di Pierro E., De Canio M., Mercadante R., Savino M., Granata F., Tavazzi D., Nicolli A.M., Trevisan A., Marchini S., Fustinoni S. (2021). Laboratory Diagnosis of Porphyria. Diagnostics.

[12] Tefferi A., Colgan J.P., Solberg L.A. (1994). Acute porphyrias: Diagnosis and management. Mayo Clin. Proc..

[13] Kou J., Dou D., Yang L. (2017). Porphyrin photosensitizers in photodynamic therapy and its applications. Oncotarget.

[14] Bandyopadhyay S., Forzano J.A., Dirak M., Chan J. (2025). Activatable Porphyrin-Based Sensors, Photosensitizers and Combination Therapeutics. JACS Au.

[15] Hasobe T. (2013). Porphyrin-Based Supramolecular Nanoarchitectures for Solar Energy Conversion. J. Phys. Chem. Lett..

[16] Magna G., Mandoj F., Stefanelli M., Pomarico G., Monti D., Di Natale C., Paolesse R., Nardis S. (2021). Recent Advances in Chemical Sensors Using Porphyrin-Carbon Nanostructure Hybrid Materials. Nanomaterials.

[17] Fagadar-Cosma E. (2018). Sensors Based on Biomimetic Porphyrin Derivatives and their Hybrid Combinations. Res. Rev. Electrochem..

[18] Jurow M., Schuckman A.E., Batteas J.D., Drain C.M. (2010). Porphyrins as Molecular Electronic Components of Functional Devices. Coord. Chem. Rev..

[19] World Health Organization (2024). Global Cancer Burden Growing, Amidst Mounting Need for Services.

[20] Bray F., Laversanne M., Sung H., Ferlay J., Siegel R.L., Soerjomataram I., Jemal A. (2024). Global cancer statistics 2022: GLOBOCAN estimates of incidence and mortality worldwide for 36 cancers in 185 countries. CA Cancer J. Clin..

[21] Vasan N., Baselga J., Hyman D.M. (2019). A view on drug resistance in cancer. Nature.

[22] Chabner B., Roberts T. (2005). Chemotherapy and the war on cancer. Nat. Rev. Cancer.

[23] Henrykowska G., Bartusik-Aebisher D., Dynarowicz K., Ramesh T.S., Smolak B., Aebisher D. (2026). Overcoming Resistance to Anti-EGFR Therapies: Mechanisms of Cetuximab and Panitumumab Resistance and Emerging Combination Strategies. Pharmaceuticals.

[24] Agostinis P., Berg K., Cengel K.A., Foster T.H., Girotti A.W., Gollnick S.O., Hahn S.M., Hamblin M.R., Juzeniene A., Kessel D. (2011). Photodynamic therapy of cancer: An update. CA Cancer J. Clin..

[25] Kwiatkowski S., Knap B., Przystupski D., Saczko J., Kędzierska E., Knap-Czop K., Kotlińska J., Michel O., Kotowski K., Kulbacka J. (2018). Photodynamic therapy-mechanisms, photosensitizers and combinations. Biomed. Pharmacother..

[26] Dougherty T.J., Gomer C.J., Henderson B.W., Jori G., Kessel D., Korbelik M., Moan J., Peng Q. (1998). Photodynamic therapy. J. Natl. Cancer Inst..

[27] Castano A.P., Mroz P., Hamblin M.R. (2006). Photodynamic therapy and anti-tumour immunity. Nat. Rev. Cancer.

[28] City of Hope (2023). [Photodynamic Therapy (PDT)]. CancerCenter.com. https://www.cancercenter.com/treatment-options/photodynamic-therapy.

[29] Dandash F., Leger D.Y., Diab-Assaf M., Sol V., Liagre B. (2021). Porphyrin/Chlorin Derivatives as Promising Molecules for Therapy of Colorectal Cancer. Molecules.

[30] Allison R.R., Downie G.H., Cuenca R., Hu X.H., Childs C.J., Sibata C.H. (2004). Photosensitizers in clinical PDT. Photodiagnosis Photodyn. Ther..

[31] Zhang Q., He J., Yu W., Li Y., Liu Z., Zhou B., Liu Y. (2020). A promising anticancer drug: A photosensitizer based on the porphyrin skeleton. RSC Med. Chem..

[32] Boscencu R., Radulea N., Manda G., Machado I.F., Socoteanu R.P., Lupuliasa D., Burloiu A.M., Mihai D.P., Ferreira L.F.V. (2023). Porphyrin Macrocycles: General Properties and Theranostic Potential. Molecules.

[33] Wang X., Lv H., Sun Y., Zu G., Zhang X., Song Y., Zhao F., Wang J. (2022). New porphyrin photosensitizers—Synthesis, singlet oxygen yield, photophysical properties and application in PDT. Spectrochim. Acta Part A Mol. Biomol. Spectrosc..

[34] Costa L.D., Silva J.D.A.e., Fonseca S.M., Arranja C.T., Urbano A.M., Sobral A.J. (2016). Photophysical Characterization and in Vitro Phototoxicity Evaluation of 5,10,15,20-Tetra(quinolin-2-yl)porphyrin as a Potential Sensitizer for Photodynamic Therapy. Molecules.

[35] Aebisher D., Serafin I., Batóg-Szczęch K., Dynarowicz K., Chodurek E., Kawczyk-Krupka A., Bartusik-Aebisher D. (2024). Photodynamic Therapy in the Treatment of Cancer—The Selection of Synthetic Photosensitizers. Pharmaceuticals.

[36] Kabiri S., Rahimi R., Mozafari M.R., Naghib S.M. (2025). Porphyrin-based metal–organic frameworks in drug delivery for cancer therapy: Promises, advances and prospects. Discov. Appl. Sci..

[37] Wang J., Zhu X., Zhang Y., Pang Z., Zhao C., Zhang F. (2025). Structural design of porous organic polymers to mitigate π-stacking-induced quenching in porphyrin/phthalocyanine photosensitizers for enhanced antibacterial activity. RSC Adv..

[38] Carter K.A., Shao S., Hoopes M.I., Luo D., Ahsan B., Grigoryants V.M., Song W., Huang H., Zhang G., Pandey R.K. (2014). Porphyrin–phospholipid liposomes permeabilized by near-infrared light. Nat. Commun..

[39] Eldem A., Tekintaş Y., Ucuncu M., Horzum N. (2025). Electrospun Nanofiber Platforms for Photodynamic Therapy: Role and Efficacy in Cancer, Antimicrobial, and Wound Healing Applications. Macromol. Mater. Eng..

[40] Gomes C.V., Costa S.M., Oliveira J.S., Calhelha R.C., Lourenço L.M.O., Fangueiro R., Ferreira D.P. (2026). Electrospun Fibrous Architectures for Localized Delivery of Photosensitizers in Cancer Therapy. Molecules.

[41] Imran M., Ramzan M., Qureshi A.K., Khan M.A., Tariq M. (2018). Emerging Applications of Porphyrins and Metalloporphyrins in Biomedicine and Diagnostic Magnetic Resonance Imaging. Biosensors.

[42] Vicente M.D., Smith K.M. (2014). Syntheses and Functionalizations of Porphyrin Macrocycles. Curr. Org. Synth..

[43] Monteiro C.J.P., Faustino M.A.F., Serpa C. (2023). Porphyrin-Based Compounds: Synthesis and Application. Molecules.

[44] Luciano M., Brückner C. (2017). Modifications of Porphyrins and Hydroporphyrins for Their Solubilization in Aqueous Media. Molecules.

[45] Rahimi H. (2026). Enhanced reactive oxygen species generation using photonic platforms containing polyethylene glycol hydrogel doped with a photosensitizer for photodynamic therapy. Photodiagnosis Photodyn. Ther..

[46] Cook L.P., Brewer G., Wong-Ng W. (2017). Structural Aspects of Porphyrins for Functional Materials Applications. Crystals.

[47] Przygoda M., Bartusik-Aebisher D., Dynarowicz K., Cieślar G., Kawczyk-Krupka A., Aebisher D. (2023). Cellular Mechanisms of Singlet Oxygen in Photodynamic Therapy. Int. J. Mol. Sci..

[48] Baptista M.S., Cadet J., Di Mascio P., Ghogare A.A., Greer A., Hamblin M.R., Lorente C., Nunez S.C., Ribeiro M.S., Thomas A.H. (2017). Type I and Type II Photosensitized Oxidation Reactions: Guidelines and Mechanistic Pathways. Photochem. Photobiol..

[49] Yudaev P., Aleksandrova Y., Chugunova E., Neganova M. (2025). The Current State of Research in the Field of Photosensitizers and Photoactivators for Photodynamic/Photothermal Cancer Therapy: A Review. Int. J. Mol. Sci..

[50] Martins W.K., Belotto R., Silva M.N., Grasso D., Suriani M.D., Lavor T.S., Itri R., Baptista M.S., Tsubone T.M. (2021). Autophagy Regulation and Photodynamic Therapy: Insights to Improve Outcomes of Cancer Treatment. Front. Oncol..

[51] Mroz P., Yaroslavsky A., Kharkwal G.B., Hamblin M.R. (2011). Cell death pathways in photodynamic therapy of cancer. Cancers.

[52] Deepak K., Shee M., Roy P.K., Mandal M. (2026). Reactive oxygen species in cell signaling and cell death: A redox perspective on metabolic and inflammatory disorders. Cell Death.

[53] Ożog Ł., Aebisher D. (2018). Singlet oxygen lifetime and diffusion measurements. Eur. J. Clin. Exp. Med..

[54] Schmittlein C. (2017). Sensors and Actuators for 2D and 3D Cell Culture Models Based on Oxygen Sensitive Culture Substrates. Doctoral Dissertation.

[55] Li E., Sun Y., Lv G., Qin F., Zhang X., Zhang Z., Zhang R., Hu Z., Cao W. (2021). The Abnormal Physicochemical Phenomena of Singlet Oxygen Sensor Green in Water in the Presence of Ultrasound. ChemistrySelect.

[56] Kim M., Hong J. (2025). A Comparative Analysis of Absorbance- and Fluorescence-Based 1,3-Diphenylisobenzofuran Assay and Its Application for Evaluating Type II Photosensitization of Flavin Derivatives. Int. J. Mol. Sci..

[57] Kumar R., Gullapalli R.R. (2024). High Throughput Screening Assessment of Reactive Oxygen Species (ROS) Generation using Dihydroethidium (DHE) Fluorescence Dye. J. Vis. Exp..

[58] Karakuzu O., Cruz M.R., Liu Y., Garsin D.A. (2019). Amplex Red Assay for Measuring Hydrogen Peroxide Production from Caenorhabditis elegans. Bio Protoc..

[59] Bancirova M. (2011). Sodium azide as a specific quencher of singlet oxygen during chemiluminescent detection by luminol and Cypridina luciferin analogues. Luminescence.

[60] Figueira F., Tomé J.P.C., Paz F.A.A. (2022). Porphyrin NanoMetal-Organic Frameworks as Cancer Theranostic Agents. Molecules.

[61] Zhang Y., Lovell J.F. (2012). Porphyrins as theranostic agents from prehistoric to modern times. Theranostics.

[62] Fagadar-Cosma E., Enache C., Dana V., Fagadar-Cosma G., Stadler H., Bazylak G. (2009). Nanostructured Glasses Based on Hybrid Silica Materials Incorporating a New Asymmetrical Phenyl-Substituted Porphyrin. Open Chem. Biomed. Methods J..

[63] Minaev B., Lindgren M. (2009). Vibration and Fluorescence Spectra of Porphyrin-Cored 2,2-Bis(methylol)-propionic Acid Dendrimers. Sensors.

[64] Owens E.A., Henary M., El Fakhri G., Choi H.S. (2016). Tissue-Specific Near-Infrared Fluorescence Imaging. Acc. Chem. Res..

[65] Teraphongphom N., Kong C., Warram J., Rosenthal E. (2017). Specimen mapping in head and neck cancer using fluorescence imaging: Specimen Mapping in HNC. Laryngoscope Investig. Otolaryngol..

[66] Abosheisha M., Nasr E., Ali M., Tamanna R., Bin Halim S., Hasan M.R., Abdelgil M., Swealem A., Ismaiel M. (2025). Near-infrared fluorescence imaging in general surgery: Applications in vascularization, tumor margin detection, and biliary anatomy. Cureus.

[67] Rabiee N., Yaraki M.T., Garakani S.M., Garakani S.M., Ahmadi S., Lajevardi A., Bagherzadeh M., Rabiee M., Tayebi L., Tahriri M. (2020). Recent advances in porphyrin-based nanocomposites for effective targeted imaging and therapy. Biomaterials.

[68] Melnik A.D., Zhebit T.S., Krylov A.B., Pukhovskaya S.G., Ivanova Y.B., Kruk M.M. (2022). Formation of J-Aggregates of 21-thia-5,10,15,20-tetra-(4-sulfonatophenyl)-porphyrin in Acidified Aqueous Solutions. J. Appl. Spectrosc..

[69] Abdelaziz B., Sarà M., Ayachi S., Zagami R., Patanè S., Romeo A., Castriciano M.A., Monsù Scolaro L. (2023). Investigation of J-Aggregates of 2,3,7,8,12,13,17,18-Octabromo-5,10,15,20-tetrakis(4-sulfonatophenyl) Porphyrin in Aqueous Solutions. Nanomaterials.

[70] Safar Sajadi S.M., Khoee S. (2021). The simultaneous role of porphyrins’ H- and J- aggregates and host-guest chemistry on the fabrication of reversible Dextran-PMMA polymersome. Sci. Rep..

[71] Ely J.O., Batt W.G. (1956). Accumulation of hematoporphyrin in animal tumors without entrance into the living cells. J. Frankl. Inst..

[72] Aebisher D., Czech S., Dynarowicz K., Misiołek M., Komosińska-Vassev K., Kawczyk-Krupka A., Bartusik-Aebisher D. (2024). Photodynamic Therapy: Past, Current, and Future. Int. J. Mol. Sci..

[73] Kwon N., Kim H., Li X., Yoon J. (2021). Supramolecular agents for combination of photodynamic therapy and other treatments. Chem. Sci..

[74] Naser I., Bartusik-Aebisher D., Smolak B., Dynarowicz K., Kowalczyk E., Guz W., Aebisher D., Henrykowska G. (2026). Photodynamic Therapy in Cancer: Mechanisms, Photosensitizer Technology, Vitamin Modulation, and Rational Combination Design. Biomedicines.

[75] Aires-Fernandes M., Botelho Costa R., Rochetti do Amaral S., Mussagy C.U., Santos-Ebinuma V.C., Primo F.L. (2022). Development of Biotechnological Photosensitizers for Photodynamic Therapy: Cancer Research and Treatment-From Benchtop to Clinical Practice. Molecules.

[76] Yang X., Palasuberniam P., Kraus D., Chen B. (2015). Aminolevulinic Acid-Based Tumor Detection and Therapy: Molecular Mechanisms and Strategies for Enhancement. Int. J. Mol. Sci..

[77] Kars M.D., Yıldırım G., Gündoğdu Y., Gönce F., Ayan E., Kılıç H.Ş. (2020). Revealing the therapeutic effects of aminolevulinate mediated femtosecond laser induced photo-chemotherapy in different cancer cells. EuroBiotech J..

[78] Garland M.J., Cassidy C.M., Woolfson D., Donnelly R.F. (2009). Designing Photosensitizers for Photodynamic Therapy: Strategies, Challenges and Promising Developments. Future Med. Chem..

[79] Richter A.M., Cerruti-Sola S., Sternberg E.D., Dolphin D., Levy J.G. (1990). Biodistribution of tritiated benzoporphyrin derivative (^3^H-BPD-MA), a new potent photosensitizer, in normal and tumor-bearing mice. J. Photochem. Photobiol. B Biol..

[80] Negri L.B., Martins T.J., da Silva R.S., Hamblin M.R. (2019). Photobiomodulation combined with photodynamic therapy using ruthenium phthalocyanine complexes in A375 melanoma cells: Effects of nitric oxide generation and ATP production. J. Photochem. Photobiol. B Biol..

[81] Oluwajembola A.M., Cleanclay W.D., Onyia A.F., Chikere B.N., Zakari S., Ndifreke E., De Campos O.C. (2024). Photosensitizers in photodynamic therapy: An advancement in cancer treatment. Results Chem..

[82] Reshetov I., Alyasova A., Shpileva O., Karalkin P., Efendiev K., Pominova D., Loschenov V., Ilyasova D., Agakina Y., Gilyadova A. (2026). Photodynamic Therapy Combined with Anticancer Drug Therapy in the Treatment of Malignant Neoplasms. Cells.

[83] Mfouo-Tynga I.S., Dias L.D., Inada N.M., Kurachi C. (2021). Features of third generation photosensitizers used in anticancer photodynamic therapy: Review. Photodiagnosis Photodyn. Ther..

[84] Alsaikhan F., Al-Saikhan T.F., Alsaikhan Y.F. (2026). Nanomedicine-Integrated Phototherapy for Cancer Theranostics: A Systematic Review of Photoresponsive Materials, Mechanisms, and Clinical Translation. Int. J. Nanomed..

[85] Machuca A., Alvarez-Fernandez Garcia R., Garcia-Garcia A., Aragoneses-Cazorla G., Castillo-Lluva S., Vallet-Regi M., González B., Garcia-Calvo E., Luque-Garcia J.L. (2026). A third-generation rhodium-based nanophotosensitizer for precision photodynamic cancer therapy. J. Colloid Interface Sci..

[86] Yu J.W., Kim D.G., Cha G.D. (2025). Multifunctional Liposomes: Smart Nanomaterials for Enhanced Photodynamic Therapy. Biomimetics.

[87] Abrahamse H., Hamblin M.R. (2016). New photosensitizers for photodynamic therapy. Biochem. J..

[88] Zhang J., Jiang C., Figueiró Longo J.P., Azevedo R.B., Zhang H., Muehlmann L.A. (2018). An updated overview on the development of new photosensitizers for anticancer photodynamic therapy. Acta Pharm. Sin. B.

[89] Yeshchenko O., Khort P., Fedotov O., Chumachenko V., Virych P., Warren H.S., Booth B.W., Bliznyuk V., Kutsevol N. (2024). Third-Generation Anticancer Photodynamic Therapy Systems Based on Star-like Anionic Polyacrylamide Polymer, Gold Nanoparticles, and Temoporfin Photosensitizer. Molecules.

[90] Dong J., Tang J., Li X., Zeng Y., Su X., He Y., Liu X. (2025). Latest developments in photosensitizers: Improving stability, specificity and responsiveness. Future Med. Chem..

[91] Zhao W., Wang L., Zhang M., Liu Z., Wu C., Pan X., Huang Z., Lu C., Quan G. (2024). Photodynamic therapy for cancer: Mechanisms, photosensitizers, nanocarriers, and clinical studies. MedComm.

[92] Park J., Lee Y.K., Park I.K., Hwang S.R. (2021). Current Limitations and Recent Progress in Nanomedicine for Clinically Available Photodynamic Therapy. Biomedicines.

[93] Li G., Wang C., Jin B., Sun T., Sun K., Wang S., Fan Z. (2024). Advances in smart nanotechnology-supported photodynamic therapy for cancer. Cell Death Discov..

[94] Choi K.Y., Liu G., Lee S., Chen X. (2012). Theranostic nanoplatforms for simultaneous cancer imaging and therapy: Current approaches and future perspectives. Nanoscale.

[95] Wang Y., Staudinger J.N., Mindt T.L., Gasser G. (2023). Theranostics with photodynamic therapy for personalized medicine: To see and to treat. Theranostics.

[96] Bian W., Wang Q., He C., Tao P., Zheng J., Zhang Y., Li J., Li F., Jia H., Ling D. (2025). A real-time cell death self-reporting theranostic agent for dynamic optimization of photodynamic therapy. Adv. Sci..

[97] Xuan L., Ju Z., Skonieczna M., Zhou P.K., Huang R. (2023). Nanoparticles-induced potential toxicity on human health: Applications, toxicity mechanisms, and evaluation models. MedComm (2020).

[98] Kim M., Shin M., Zhao Y., Ghosh M., Son Y.O. (2024). Transformative Impact of Nanocarrier-Mediated Drug Delivery: Overcoming Biological Barriers and Expanding Therapeutic Horizons. Small Sci..

[99] Parvin N., Aslam M., Alam M.N., Mandal T.K. (2025). Nanotechnology Driven Innovations in Modern Pharmaceutics: Therapeutics, Imaging, and Regeneration. Nanomaterials.

[100] Akbar A., Khan S., Chatterjee T., Ghosh M. (2023). Unleashing the power of porphyrin photosensitizers: Illuminating breakthroughs in photodynamic therapy. J. Photochem. Photobiol. B Biol..

[101] Abbas M. (2026). Metal-Organic Frameworks: Porous Materials and Functional Platforms.

[102] Chen J., Zhu Y., Kaskel S. (2021). Porphyrin-Based Metal-Organic Frameworks for Biomedical Applications. Angew. Chem. Int. Ed. Engl..

[103] Shee N.K., Kim H.J. (2024). Recent Developments in Porphyrin-Based Metal-Organic Framework Materials for Water Remediation under Visible-Light Irradiation. Int. J. Mol. Sci..

[104] Yu W., Zhen W., Zhang Q., Li Y., Luo H., He J., Liu Y. (2020). Porphyrin-Based Metal-Organic Framework Compounds as Promising Nanomedicines in Photodynamic Therapy. ChemMedChem.

[105] Behera D., Priyadarshini P., Parida K. (2025). ZIF-8 metal-organic frameworks and their hybrid materials: Emerging photocatalysts for energy and environmental applications. Dalton Trans..

[106] Wang J., Fan Y., Tan Y., Zhao X., Zhang Y., Cheng C., Yang M. (2018). Porphyrinic Metal–Organic Framework PCN-224 Nanoparticles for Near-Infrared-Induced Attenuation of Aggregation and Neurotoxicity of Alzheimer’s Amyloid-β Peptide. ACS Appl. Mater. Interfaces.

[107] Liu S., Meng Q., Liu Z., Wang J., Li J., Ma X., Hu Y., Wang Z., Ma P., Lin J. (2025). Engineered Metal–Organic Framework with Stereotactic Anchoring and Spatial Separation of Porphyrins for Amplified Ultrasound-Mediated Pyroptosis and Cancer Immunotherapy. Angew. Chem. Int. Ed..

[108] Guan L., Liu F., Zhang C., Wang W., Zhang J., Liang Q. (2024). Porphyrin-based metal-organic frameworks for cancer theranostics. Appl. Surf. Sci. Adv..

[109] Ghosh R., Hasan M.S., Gothwal S., Lee J., Dhasaiyan P., Ryu J.-H. (2026). Stimuli-Responsive Zirconium-Based Metal–Organic Frameworks for Targeted Cancer Drug Delivery. ACS Appl. Mater. Interfaces.

[110] Zhu X., Liao M., Wei H., Jiang Y., Cheng H., Xu X., Xu S., Hu Y., Chai R., Qian X. (2026). Advances and challenges of metal-organic frameworks in controlled drug delivery. Mater. Today Bio.

[111] Wang M., Cheng Y., Zhang M., Liu S., Luo L., Han X., Chen J., Xia Z., Luo H. (2026). Multifunctional MOF composites for advanced drug identification and screening: Innovations, applications and challenges. J. Pharm. Anal..

[112] ACS Material LLC (2017). Introduction to MOFs & COFs.

[113] CAS (2025). Covalent Organic Frameworks (COFs) are Driving Breakthroughs in Renewable Energy and Biomedicine. https://www.cas.org/resources/cas-insights/covalent-organic-frameworks.

[114] Magadla A. (2025). Hybrid Nanoplatforms Based on Photosensitizers and Metal/Covalent Organic Frameworks for Improved Cancer Synergistic Treatment Nano-Delivery Systems. Molecules.

[115] Sharma R., Yadav P., Yadav M., Gupta R., Rana P., Srivastava A., Zboril R., Varma R., Antonietti M., Gawande M. (2019). Recent Development of Covalent Organic Frameworks (COFs): Synthesis and Catalytic (Organic-Electro-Photo) Applications. Mater. Horiz..

[116] Sheng Y., Ma K. (2026). Exploration of covalent-organic frameworks and metal-organic frameworks for drug delivery applications. Front. Chem..

[117] Tripathy D.B., Pradhan S., Agarwal P. (2026). Toxicity of metal-organic frameworks (MOFs): Mini review. Proc. Inst. Mech. Eng. Part N. J. Nanomater. Nanoeng. Nanosyst..

[118] Xue J., Zhuang J., Fan T., Tang S., Wang J., Liu X., Liu J., Shi J., Li J., Li W. (2026). Redox-responsive tellurium-bridged covalent organic frameworks/PEG composites for targeted therapy of diabetic cardiomyopathy. Adv. Sci..

[119] Chen P., Li Y., Dai Y., Wang Z., Zhou Y., Wang Y., Li G. (2024). Porphyrin-based covalent organic frameworks as doxorubicin delivery system for chemo-photodynamic synergistic therapy of tumors. Photodiagnosis Photodyn. Ther..

[120] Qi C., Chen J., Qu Y., Luo X., Wang W., Zheng X. (2024). Recent Advances in Porphyrin-Based Covalent Organic Frameworks for Synergistic Photodynamic and Photothermal Therapy. Pharmaceutics.

[121] Qu Y., Li K., Du X., Jia H., Xu S., Zhang M. (2026). Review of research progress of porphyrin functionalized COFs in photodynamic therapy. Appl. Mater. Today.

[122] Zhang Y., Zhang M., Hu X., Hao H., Quan C., Ren T., Gao H., Wang J. (2024). Engineering a porphyrin COFs encapsulated by hyaluronic acid tumor-targeted nanoplatform for sequential chemo-photodynamic multimodal tumor therapy. Int. J. Biol. Macromol..

[123] Zou Y., Chen M., Chen J., Wang W., Zheng X. (2026). Covalent organic frameworks for cancer immunotherapy: Mechanisms, applications, and prospects. Front. Immunol..

[124] Irie T., Das S., Negishi Y. (2026). Covalent organic frameworks as precision nanocarriers for targeted drug delivery: Developments, hurdles, and horizons. Nanoscale Horiz..

[125] Kolodziejczyk J., Plonska-Brzezinska M.E. (2026). Potential of Covalent Organic Framework in Pharmacy and Biomedicine: Influence of Structure on Properties and Applications. ChemMedChem.

[126] Arai T., Tanaka M., Kawakami H. (2012). Porphyrin-Containing Electrospun Nanofibers: Positional Control of Porphyrin Molecules in Nanofibers and Their Catalytic Application. ACS Appl. Mater. Interfaces.

[127] Zhou W., Jiang Z., Lin X., Chen Y., Wu Q., Chen J., Zhang F., Xie G., Zhang Y., Lin J. (2024). Preparation of MPN@Zein-PpIX Membrane and Its Antibacterial Properties. ACS Omega.

[128] Casula L., Adeela Y., Lai F., Bradić J., Schlich M., Dragićević N., Sinico C. (2026). Healing the wound with nanomedicine: The therapeutic potential of liposomes and nanofibers. J. Drug Deliv. Sci. Technol..

[129] Costa S.M., Fangueiro R., Ferreira D.P. (2022). Drug Delivery Systems for Photodynamic Therapy: The Potentiality and Versatility of Electrospun Nanofibers. Macromol. Biosci..

[130] Rajabifar N., Rostami A., Afshar S., Mosallanezhad P., Zarrintaj P., Shahrousvand M., Nazockdast H. (2024). Wound Dressing with Electrospun Core-Shell Nanofibers: From Material Selection to Synthesis. Polymers.

[131] Altıntaş S. Green Manufacturing of Core-Shell Polyvinyl Alcohol-Gelatin Electrospun Nanofiber Scaffolds. Proceedings of the 2nd World Congress on Recent Advances in Nanotechnology.

[132] Liu Y., Li X., Li L., Liu Z., Zhao C., Jiao T. (2024). Recent progress in gelatin-based electrospun nanofibrous membranes for wound dressings: A Review. J. Mol. Liq..

[133] Jiffrin R., Razak S.I.A., Jamaludin M.I., Hamzah A.S.A., Mazian M.A., Jaya M.A.T., Nasrullah M.Z., Majrashi M., Theyab A., Aldarmahi A.A. (2022). Electrospun Nanofiber Composites for Drug Delivery: A Review on Current Progresses. Polymers.

[134] Zulkifli M.Z.A., Nordin D., Shaari N., Kamarudin S.K. (2023). Overview of Electrospinning for Tissue Engineering Applications. Polymers.

[135] Mendes A.C., Moura N.M.M., Faustino M.A.F., Neves M.G.P.M.S., Chronakis I.S. (2025). Porphyrin-based electrospun nanomaterials for life science applications. Biophys. Rev..

[136] Banu N., de la Rosa E., Saeed M.A., Salas P., Panikar S.S. (2026). Liposome-Based Photodynamic Therapy for Breast Cancer: Innovations in Targeted Delivery, Combination Strategies, and Clinical Translation. Int. J. Mol. Sci..

[137] Lovell J. (2023). Porphyrin-phospholipid liposomes and chemophototherapy. Photodiagnosis Photodyn. Ther..

[138] Vagena I.-A., Malapani C., Gatou M.-A., Lagopati N., Pavlatou E.A. (2025). Enhancement of EPR Effect for Passive Tumor Targeting: Current Status and Future Perspectives. Appl. Sci..

[139] Belyaev I.B., Griaznova O.Y., Yaremenko A.V., Deyev S.M., Zelepukin I.V. (2025). Beyond the EPR effect: Intravital microscopy analysis of nanoparticle drug delivery to tumors. Adv. Drug Deliv. Rev..

[140] Mattheolabakis G., Milane L., Singh A., Amiji M.M. (2015). Hyaluronic acid targeting of CD44 for cancer therapy: From receptor biology to nanomedicine. J. Drug Target..

[141] Feng Q., Wang J., Song H., Zhuo L.-G., Wang G., Liao W., Feng Y., Wei H., Chen Y., Yang Y. (2018). Uptake and light-induced cytotoxicity of hyaluronic acid-grafted liposomes containing porphyrin in tumor cells. J. Drug Deliv. Sci. Technol..

[142] Miyazaki M., Yuba E., Hayashi H., Harada A., Kono K. (2018). Hyaluronic Acid-Based pH-Sensitive Polymer-Modified Liposomes for Cell-Specific Intracellular Drug Delivery Systems. Bioconjugate Chem..

[143] Lee Y., Thompson D.H. (2017). Stimuli-responsive liposomes for drug delivery. Wiley Interdiscip. Rev. Nanomed. Nanobiotechnol..

[144] Siriwardane D.A., Jiang W., Mudalige T. (2024). Profiling in-vitro release of verteporfin from VISUDYNE^®^ liposomal formulation and investigating verteporfin binding to human serum albumin. Int. J. Pharm..

[145] Valeant Canada LP (2018). VISUDYNE^®^ (Verteporfin for Injection): Product Monograph. [Control number: 208530]. https://pdf.hres.ca/dpd_pm/00043080.PDF.

[146] CHEPLAPHARM Arzneimittel GmbH (2024). Visudyne^®^ 15 mg Powder for Solution for Infusion: Summary of Product Characteristics.

[147] Lu J., Roy B., Anderson M., Leggett C.L., Levy M.J., Pogue B., Hasan T., Wang K.K. (2019). Verteporfin- and sodium porfimer-mediated photodynamic therapy enhances pancreatic cancer cell death without activating stromal cells in the microenvironment. J. Biomed. Opt..

[148] Stevanović M.M., Qian K., Huang L., Vukomanović M. (2025). PLGA-Based Co-Delivery Nanoformulations: Overview, Strategies, and Recent Advances. Pharmaceutics.

[149] Fong S.S., Foo Y.Y., Saw W.S., Leo B.F., Teo Y.Y., Chung I., Goh B.T., Misran M., Imae T., Chang C.C. (2022). Chitosan-Coated-PLGA Nanoparticles Enhance the Antitumor and Antimigration Activity of Stattic—A STAT3 Dimerization Blocker. Int. J. Nanomed..

[150] Najm A., Niculescu A.-G., Bolocan A., Rădulescu M., Grumezescu A.M., Beuran M., Gaspar B.S. (2024). Chitosan and Cyclodextrins—Versatile Materials Used to Create Drug Delivery Systems for Gastrointestinal Cancers. Pharmaceutics.

[151] Du J., Jin C., Dang Z., Ma R., Chen C., Bian Q., Lv P., Ji Y., Gao W., Liu S. (2026). Based on polysaccharide nanocarriers: Mechanisms of bioactivity potentiation and latest research progress. Front. Biomater. Sci..

[152] Mura P.A. (2026). Advantages of the Combined Use of Cyclodextrins and Chitosan in Drug Delivery: A Review. Pharmaceutics.

[153] Has C. (2026). PEG Alternatives in Liposomal Nanocarriers: Physicochemical Characteristics and Sustainable Process Strategies. Asia-Pac. J. Chem. Eng..

[154] Xu Z., Xie Y., Chen W., Deng W. (2025). Nanocarrier-Based Systems for Targeted Delivery: Current Challenges and Future Directions. MedComm (2020).

[155] Hu J., Hu J., Wu W., Qin Y., Fu J., Liu C., Seeberger P.H., Yin J. (2022). Bimodal treatment of hepatocellular carcinoma by targeted minimally interventional photodynamic/chemotherapy using glyco-covalent-organic frameworks-guided porphyrin/sorafenib. Acta Biomater..

[156] Qian J.J., Guo J.X., Wang M.C., Chen L.J., Zhao X., Yan X.P. (2025). Cationic porphyrin-based covalent organic frameworks for enhanced phototherapy and targeted chemotherapy of bacterial infections. J. Colloid Interface Sci..

[157] Sun Q., Bi H., Wang Z., Li C., Wang C., Xu J., Yang D., He F., Gai S., Yang P. (2019). O2 Generating Metal–Organic Framework-Based Hydrophobic Photosensitizer Delivery System for Enhanced Photodynamic Therapy. ACS Appl. Mater. Interfaces.

[158] Sun X., Chen K., Liu Y., Zhang G., Shi M., Shi P., Zhang S. (2021). Metal–organic framework combined with CaO2 nanoparticles for enhanced and targeted photodynamic therapy. Nanoscale Adv..

[159] Costa S.M., Lourenço L.M.O., Calhelha R.C., Calejo I., Barrias C.C., Fangueiro R., Ferreira D.P. (2024). Localized cancer photodynamic therapy approach based on core–shell electrospun nanofibers. Mater. Adv..

[160] de Oliveira D.C.S., de Freitas C.F., Calori I.R., Goncalves R.S., Cardinali C.A.E.F., Malacarne L.C., Ravanelli M.I., de Oliveira H.P.M., Tedesco A.C., Caetano W. (2020). Theranostic verteporfin- loaded lipid-polymer liposome for photodynamic applications. J. Photochem. Photobiol. B.

[161] Fu X., Yang Z., Deng T., Chen J., Wen Y., Fu X., Zhou L., Zhu Z., Yu C. (2020). A natural polysaccharide mediated MOF-based Ce6 delivery system with improved biological properties for photodynamic therapy. J. Mater. Chem. B.

[162] Xu J., Lai Y., Wang F., Zou Z., Pei R., Yu H., Xu Z. (2023). Dual stimuli-activatable versatile nanoplatform for photodynamic therapy and chemotherapy of triple-negative breast cancer. Chin. Chem. Lett..

[163] Son J., Yang S.M., Yi G., Roh Y.J., Park H., Park J.M., Choi M.G., Koo H. (2018). Folate-modified PLGA nanoparticles for tumor-targeted delivery of pheophorbide a in vivo. Biochem. Biophys. Res. Commun..

[164] Savellano M.D., Pogue B.W., Hoopes P.J., Vitetta E.S., Paulsen K.D. (2005). Multiepitope HER2 Targeting Enhances Photoimmunotherapy of HER2-Overexpressing Cancer Cells with Pyropheophorbide-a Immunoconjugates. Cancer Res..

[165] Lou J., Aragaki M., Bernards N., Chee T., Gregor A., Hiraishi Y., Ishiwata T., Leung C., Ding L., Kitazawa S. (2023). Repeated photodynamic therapy mediates the abscopal effect through multiple innate and adaptive immune responses with and without immune checkpoint therapy. Biomaterials.

[166] Ren Q., Sheng Y., Tao C., Niu S., Yu N., Chen Z., Lian W. (2024). Zinc peroxide-based nanotheranostic platform with endogenous hydrogen peroxide/oxygen generation for enhanced photodynamic-chemo therapy of tumors. J. Colloid Interface Sci..

[167] Ramos Silva C., Berionni G., Simões Ribeiro M., Heuskin A.-C. (2026). Porphyrin-coated gold nanoparticles associated with X-rays and proton therapy in the treatment of triple-negative breast cancer. Nanoscale Adv..

[168] Correia J.H., Rodrigues J.A., Pimenta S., Dong T., Yang Z. (2021). Photodynamic Therapy Review: Principles, Photosensitizers, Applications, and Future Directions. Pharmaceutics.

[169] Zou Y., Zhao Y., Yu J., Luo X., Han J., Ye Z., Li J., Lin H. (2016). Photodynamic therapy versus surgical excision to basal cell carcinoma: Meta-analysis. J. Cosmet. Dermatol..

[170] Pucelik B., Barzowska A., Sułek A., Werłos M., Dąbrowski J.M. (2024). Refining antimicrobial photodynamic therapy: Effect of charge distribution and central metal ion in fluorinated porphyrins on effective control of planktonic and biofilm bacterial forms. Photochem. Photobiol. Sci..

[171] Amos-Tautua B., Songca S., Oluwafemi O. (2019). Application of Porphyrins in Antibacterial Photodynamic Therapy. Molecules.

[172] Liu Y., Qin R., Zaat S.A.J., Breukink E., Heger M. (2015). Antibacterial photodynamic therapy: Overview of a promising approach to fight antibiotic-resistant bacterial infections. J. Clin. Transl. Res..

[173] Overchuk M., Weersink R.A., Wilson B.C., Zheng G. (2023). Photodynamic and Photothermal Therapies: Synergy Opportunities for Nanomedicine. ACS Nano.

[174] Xie M., Gong T., Wang Y., Li Z., Lu M., Luo Y., Min L., Tu C., Zhang X., Zeng Q. (2024). Advancements in Photothermal Therapy Using Near-Infrared Light for Bone Tumors. Int. J. Mol. Sci..

[175] Kong C., Chen X. (2022). Combined Photodynamic and Photothermal Therapy and Immunotherapy for Cancer Treatment: A Review. Int. J. Nanomed..

[176] Trinidad A.J., Hong S.J., Peng Q., Madsen S.J., Hirschberg H. (2014). Combined concurrent photodynamic and gold nanoshell loaded macrophage-mediated photothermal therapies: An in vitro study on squamous cell head and neck carcinoma. Lasers Surg. Med..

[177] Cressey P., Abd Shukor S.B., Thanou M. (2025). Sonodynamic therapy: Transforming sound into light for hard-to-treat tumours. Adv. Drug Deliv. Rev..

[178] Liu R., Zhang Q., Lang Y., Peng Z., Li L. (2017). Sonodynamic therapy, a treatment developing from photodynamic therapy. Photodiagnosis Photodyn. Ther..

[179] Ponce Ayala E.T., Alves Dias de Sousa F., Vollet-Filho J.D., Rodrigues Garcia M., de Boni L., Salvador Bagnato V., Pratavieira S. (2021). Photodynamic and sonodynamic therapy with protoporphyrin IX: In vitro and in vivo studies. Ultrasound Med. Biol..

[180] Wang Y., Zhang Y., Zhang X., Zhang Z., She J., Wu D., Gao W. (2022). High Drug-Loading Nanomedicines for Tumor Chemo-Photo Combination Therapy: Advances and Perspectives. Pharmaceutics.

[181] Crescenzi E., Chiaviello A., Canti G., Reddi E., Veneziani B.M., Palumbo G. (2006). Low doses of cisplatin or gemcitabine plus Photofrin/photodynamic therapy: Disjointed cell cycle phase-related activity accounts for synergistic outcome in metastatic non–small cell lung cancer cells (H1299). Mol. Cancer Ther..

[182] Zhao Y., Liu X., Liu X., Yu J., Bai X., Wu X., Guo X., Liu Z., Liu X. (2022). Combination of phototherapy with immune checkpoint blockade: Theory and practice in cancer. Front. Immunol..

[183] Zhao R., Li S., Zhao J., Yao C. (2025). Advancements in Nano-Delivery Systems for Photodynamic and Photothermal Therapy to Induce Immunogenic Cell Death in Tumor Immunotherapy. Int. J. Nanomed..

[184] Thiruppathi J., Vijayan V., Park I.K., Lee S.E., Rhee J.H. (2024). Enhancing cancer immunotherapy with photodynamic therapy and nanoparticle: Making tumor microenvironment hotter to make immunotherapeutic work better. Front. Immunol..

[185] Chang Y., Lim Y.G., Park K. (2025). Synergistic photodynamic and chemodynamic therapy using hypoxia-adaptive Ce6@Co nanoparticles for enhanced tumor suppression. Int. J. Pharm. X.

[186] Han S., Ninjbadgar T., Kang M., Kim C., Kim J. (2023). Recent Advances in Photoacoustic Agents for Theranostic Applications. Nanomaterials.

[187] Neprokin A., Broadway C., Myllylä T., Bykov A., Meglinski I. (2022). Photoacoustic Imaging in Biomedicine and Life Sciences. Life.

[188] Pyrzynska K., Kilian K., Pęgier M. (2022). Porphyrins as Chelating Agents for Molecular Imaging in Nuclear Medicine. Molecules.

[189] Luo Y., Zeng Z., Shan T., Xu X., Chen J., He Y., Zhang T., Huang Z., Chai G., Huang Y. (2022). Fibroblast activation protein α activatable theranostic pro-photosensitizer for accurate tumor imaging and highly-specific photodynamic therapy. Theranostics.

[190] Cramers P., Ruevekamp M., Oppelaar H., Dalesio O., Baas P., Stewart F.A. (2003). Foscan uptake and tissue distribution in relation to photodynamic efficacy. Br. J. Cancer.

[191] Nitta M., Muragaki Y. (2017). P09.37 Photodynamic therapy using Talaporfin sodium and semiconductor laser combined with maximum tumor resection improves prognosis of patients with glioblastoma. Neuro Oncol..

[192] Baskaran R., Lee J., Yang S.G. (2018). Clinical development of photodynamic agents and therapeutic applications. Biomater. Res..

[193] Lipka B., Lipka M., Rycerski M., Toroń M., Wołoszyn A., Tokarczyk W., Mordarski J. (2026). Current landscape of photosensitizers in photodynamic therapy: Challenges and future perspectives—A literature review. Wiad. Lek..

[194] Roberts W., Smith K., Mcculiough J., Berns M. (2008). Skin Photosensitivity and Photodestruction of Several Potential Photodynamic Sensitizers. Photochem. Photobiol..

[195] Cai Y., Chai T., Nguyen W., Liu J., Xiao E., Ran X., Ran Y., Du D., Chen W., Chen X. (2025). Phototherapy in cancer treatment: Strategies and challenges. Signal Transduct. Target. Ther..

[196] Yu L., Liu Z., Xu W., Jin K., Liu J., Zhu X., Zhang Y., Wu Y. (2024). Towards overcoming obstacles of type II photodynamic therapy: Endogenous production of light, photosensitizer, and oxygen. Acta Pharm. Sin. B.

[197] Hong L., Li J., Luo Y., Guo T., Zhang C., Ou S., Long Y., Hu Z. (2022). Recent Advances in Strategies for Addressing Hypoxia in Tumor Photodynamic Therapy. Biomolecules.

[198] Chauhan D.S., Prasad R., Dhanka M., Kaur N., Vithalani H., Liyanapathirana K., Nanjundappa R.H., Gao H., Umeshappa C.S. (2026). Nanoparticles-based phototherapy systems: Molecular mechanisms and clinical applications. Signal Transduct. Target. Ther..

[199] Li T., Sun W., Pan H., Tang Q., Geng L., Liu J., Liao G., Zhang Y. (2026). Advancing biomedical technology through multifunctional porphyrin-based MOFs: Design principles, applications, and biosafety evaluations. Mater. Today Bio.

[200] Đorđević S., Gonzalez M.M., Conejos-Sánchez I., Carreira B., Pozzi S., Acúrcio R.C., Satchi-Fainaro R., Florindo H.F., Vicent M.J. (2022). Current hurdles to the translation of nanomedicines from bench to the clinic. Drug Deliv. Transl. Res..

[201] Qu C., Wu F., Shen Z. (2026). Molecular Engineering of Porphyrins for Near-Infrared Bioimaging and Phototherapy. Chem. Biomed. Imaging.

[202] Wang T., Chen Y., Wang B., Gao X., Wu M. (2022). Recent Progress in Second Near-Infrared (NIR-II) Fluorescence Imaging in Cancer. Biomolecules.

[203] Xiang D., Wang Z., Zheng H., Tang Y., Li Q. (2026). Organic small-molecule NIR-II fluorophores for tumor phototheranostics. Light Sci. Appl..

[204] Zou Y., Chen J., Luo X., Qu Y., Zhou M., Xia R., Wang W., Zheng X. (2024). Porphyrin-engineered nanoscale metal-organic frameworks: Enhancing photodynamic therapy and ferroptosis in oncology. Front. Pharmacol..

[205] Yang P., Zhang J., Chang Y., Tang L., Ma G., Liu X., Gao F., Ma X., Guo Y. (2025). Tumor microenvironment responsive smart nanoplatform for synergistic tumor therapy through co-enhancement of GSH depletion and hypoxia relief. J. Inorg. Biochem..

[206] Park W., Cho S., Han J., Shin H., Na K., Lee B., Kim D.H. (2017). Advanced smart-photosensitizers for more effective cancer treatment. Biomater. Sci..

[207] Warszyńska M., Repetowski P., Dąbrowski J.M. (2023). Photodynamic therapy combined with immunotherapy: Recent advances and future research directions. Coord. Chem. Rev..

[208] Lin Y., Xie R., Yu T. (2024). Photodynamic Therapy for Atherosclerosis: Past, Present, and Future. Pharmaceutics.

[209] Josefsen L.B., Boyle R.W. (2012). Unique diagnostic and therapeutic roles of porphyrins and phthalocyanines in photodynamic therapy, imaging and theranostics. Theranostics.

